# Machine learning–driven discovery of antimicrobial peptides against *Pseudomonas aeruginosa*

**DOI:** 10.3389/fphar.2026.1837055

**Published:** 2026-06-24

**Authors:** YaTong Zhang, Xuelin Sun, HuiBo Li, DanDan Li, Caiyuan Yu, Bin Zhao, YingChen Zhou, Yi Zhun Zhu, Rongsheng Zhao

**Affiliations:** 1 Faculty of Medicine, School of Pharmacy, Macau University of Science and Technology, Taipa, Macau, China; 2 Department of Pharmacy, Beijing Hospital, National Center of Gerontology; Institute of Geriatric Medicine, Chinese Academy of Medical Sciences, Beijing, China; 3 Department of Pharmacy, Peking University Third Hospital, Beijing, China; 4 Institute for Drug Evaluation, Peking University Health Science Center, Beijing, China; 5 Department of Pharmacy, Beijing Friendship Hospital, Capital Medical University, Beijing, China; 6 College of Agroforestry and Medicine, The Open University of China, Beijing, China; 7 Department of Pharmacy, Peking Union Medical College, Chinese Academy of Medical Sciences, Beijing, China; 8 Shanghai Key Laboratory of Bioactive Small Molecules, Department of Pharmacology, School of Pharmacy, Fudan University, Shanghai, China

**Keywords:** antibacterial mechanism, antimicrobial peptides, machine learning, molecular dynamics simulation, transcriptomics

## Abstract

**Introduction:**

Antibiotic resistance has become a global health crisis, driving the urgent need for novel antibacterial strategies. Antimicrobial peptides (AMPs) have emerged as promising therapeutic candidates due to their broad-spectrum activity and low propensity for resistance development. However, conventional discovery approaches remain time-consuming and inefficient for large-scale screening.

**Methods:**

In this study, we developed an integrated prediction framework that combines deep learning with classical machine learning methods to enable rapid and accurate identification of AMPs. The model was applied to screen over 250,000 artificially generated peptide sequences. Candidate peptides were selected based on physicochemical descriptors and activity-associated features, followed by solid-phase synthesis and minimum inhibitory concentration assays against Pseudomonas aeruginosa. Mechanistic investigations were conducted using scanning electron microscopy to visualize membrane damage, molecular dynamics simulations to probe peptide-membrane interactions, and transcriptomic profiling to assess bacterial stress responses and metabolic pathway alterations.

**Results:**

Ten promising antibacterial candidates were successfully identified and experimentally validated. All selected peptides exhibited measurable activity against P. aeruginosa, with several showing potent inhibitory effects. Microscopic and simulation analyses confirmed that the peptides exert their antibacterial effects primarily through membrane disruption. Transcriptomic data further revealed that these peptides interfere with key metabolic pathways and activate bacterial stress response systems.

**Discussion:**

Our findings demonstrate that the integrated deep learning-machine learning pipeline offers an efficient and reliable approach for large-scale AMP discovery. The mechanistic insights gained from this study not only validate the predicted candidates but also provide a foundation for rational optimization of peptide-based therapeutics. This comprehensive strategy holds promise for accelerating the development of next-generation alternatives to conventional antibiotics.

## 1 Introduction

Bacterial infectious diseases remain a major threat to global public health and currently represent the second leading cause of death worldwide. A comprehensive analysis published in *The Lancet* reported that in 2019 approximately 7.7 million deaths were directly attributable to 33 major bacterial pathogens, exceeding the combined mortality associated with HIV and malaria during the same period (Vos et al., 2020). Among these organisms, *Staphylococcus aureus*, *Escherichia coli*, *Streptococcus pneumoniae*, *Klebsiella pneumoniae*, and *Pseudomonas aeruginosa* were responsible for over half of the reported deaths. Despite the significant reduction in infection-related mortality achieved through extensive antibiotic application, the improper and excessive use of these drugs has driven the rapid emergence of antimicrobial resistance, which has become one of the most critical medical challenges of the twenty-first century (Yu et al., 2018).

Antibiotic resistance develops as pathogenic bacteria rapidly adapt to pharmacological selective pressures through diverse evolutionary strategies, such as horizontal gene exchange, biofilm development, and metabolic reprogramming (Kok et al., 2022; Rather et al., 2021; Yang et al., 2024). Previous research has shown that environmental stressors can promote the formation of resistant variants via coordinated chromosomal mutations and plasmid-mediated gene transfer (Yang et al., 2024). Moreover, biofilms create dense extracellular polymer matrices that hinder antimicrobial penetration while enhancing the spread of resistance determinants, thereby substantially diminishing antibiotic effectiveness (Liu et al., 2024; Rather et al., 2021). Beyond classical resistance mechanisms, metabolic remodeling has emerged as an important adaptive strategy, allowing bacteria to counteract antibiotic-induced damage by reshaping intracellular metabolic pathways (Lopatkin et al., 2021). Of particular concern, the global propagation of resistance genes such as NDM-1 has severely undermined the efficacy of last-line therapies, including carbapenems (Khan et al., 2017), and the extensive use of antibiotics in animal husbandry has further facilitated resistance transmission across environmental reservoirs and human populations (Inda-Díaz et al., 2023; Manyi-Loh et al., 2018). Together, these trends underscore the urgent need for innovative antimicrobial approaches that maintain therapeutic effectiveness while minimizing the risk of resistance development.

*Pseudomonas aeruginosa* is a widely distributed Gram-negative microorganism commonly found in human-associated niches and is recognized as an opportunistic pathogen responsible for numerous clinical infections (Rutherford et al., 2018). It contributes to a wide range of serious diseases, including respiratory tract infections, urinary system infections, septicemia, as well as infections associated with burns and chronic wounds (Igídio et al., 2025). Within wound environments, this bacterium readily adapts to moist and low-oxygen conditions, where it establishes persistent biofilms that substantially impede tissue repair processes and intensify tissue injury by releasing virulence determinants and triggering prolonged inflammatory reactions (Verhoeve et al., 2022). Furthermore, both inherent and acquired resistance to diverse antimicrobial agents severely complicates therapeutic intervention.

Antimicrobial peptides are small bioactive molecules synthesized by various organisms that inhibit microbial growth primarily through membrane disruption or interference with vital cellular functions, making them attractive alternatives for combating antibiotic-resistant pathogens (Mahlapuu et al., 2016). Nevertheless, conventional discovery pipelines for these peptides rely largely on experimental screening, which is time-consuming and financially demanding (Rivero-Pino et al., 2023). The rapid expansion of antimicrobial peptide repositories has facilitated the development of artificial intelligence–driven predictive systems for peptide identification.

In recent years, the integration of artificial intelligence has profoundly revolutionized the landscape of antimicrobial peptide discovery, transcending the limitations of traditional experimental methodologies (Li et al., 2026; Yang et al., 2026). The field has transitioned from early computational frameworks that primarily combined classical machine learning models such as Support Vector Machines (SVM) and Random Forests (RF) with handcrafted physicochemical and compositional descriptors to more advanced deep learning architectures (Bournez et al., 2023; Joseph et al., 2012; Sharma et al., 2021). By integrating natural language processing techniques, recent deep learning models have achieved superior predictive accuracy through the autonomous extraction of complex sequence features (Du et al., 2023; Ma et al., 2022; Xu et al., 2023). Furthermore, the emergence of protein based Large Language Models (LLMs) represents a landmark breakthrough in this domain. Models such as ESM-2 possess the capability to extract high-level contextual representations from massive protein sequence datasets, providing innovative feature embeddings for AMP activity prediction (Yuan et al., 2023). These models effectively capture the biological language of peptides and encode structural and functional information into dense embedding vectors without the requirement for prior feature engineering. Despite their immense potential, systematic studies that integrate ESM-2 with diverse machine learning strategies for AMP screening remain relatively scarce. Consequently, there is a compelling need to further evaluate the performance compatibility of various computational algorithms with ESM-2 representations to enhance the overall efficiency of peptide discovery.

Meanwhile, advances in high-throughput sequencing have established bacterial transcriptomics as a robust tool for characterizing cellular physiological responses and reconstructing regulatory networks. Through comparative analysis of gene expression under distinct experimental conditions, global molecular pathways responding to external perturbations can be revealed (Jhong et al., 2019). Consequently, transcriptome-based approaches have been extensively employed to clarify the modes of action of antimicrobial peptides (Ju et al., 2025; Shan et al., 2024). Such analyses enable the identification of peptide-induced regulatory changes affecting membrane integrity, metabolic functions, and stress adaptation mechanisms, thereby offering mechanistic insight into antimicrobial efficacy and potential resistance development.

In the present work, we integrated ESM-2 representations with multiple machine learning algorithms to develop and optimize a high-accuracy antibacterial peptide prediction framework. This platform facilitated large-scale virtual screening of more than 250,000 peptide candidates, followed by experimental verification of selected sequences through solid-phase synthesis. Moreover, scanning electron microscopy and molecular dynamics simulations were applied to characterize membrane disruption behavior and interaction mechanisms of the most effective peptides against *Pseudomonas aeruginosa*, while transcriptomic profiling was employed to comprehensively decipher the inhibitory molecular responses. Overall, this study establishes an efficient computational–experimental pipeline for antimicrobial peptide discovery and provides mechanistic foundations supporting the development of novel antibiotic alternatives.

## 2 Materials and methods

### 2.1 Materials

The ten peptide candidates investigated in this work, named AP1 through AP10, possessed the amino acid sequences LNVLKRVKVEQRLNLF, GLTLKHLKKLIF, LVNKLKSVLAKY, IIKLILKHNKLAKIEY, VKKLRLEKVVLY, LSKNQKVLLRLF, RVSSK-LRLLTLF, RKVRGPPRIWVIWRR, MAKYRIRI, and FRVVWGRRGL, respectively. These peptides were prepared in our laboratory using solid-phase peptide synthesis, with puri-ty >95%. Fmoc-protected amino acids and Rink Amide MBHA resin were supplied by Shanghai Gill Biochemical Co., Ltd. Nisin Z (≥1000 IU mg^-1^), purchased from Aladdin Bi-ochemical Technology Co., Ltd. (Shanghai, China), served as the positive control. All bac-terial cultures were maintained in Luria–Bertani (LB) broth at 37 °C.

### 2.2 Data collection and model construction

Datasets employed for developing the antimicrobial peptide (AMP) prediction system were obtained from previously reported sources (Chen et al., 2024; Ma et al., 2022; Xu et al., 2023). We collected positive and negative sample datasets from previous studies and used CD-Hit to remove highly similar sequences (with a similarity of > 0.75). A total of 10,010 peptides were collected, with the ratio of antibacterial peptides to non-antibacterial peptides being 1:1. Subsequently, we randomly extracted 80% of the data from the positive and negative samples as the training set, and the remaining 20% as the test set. During the training process, the training set adopted stratified sampling - 10-fold cross-validation. In addition, we have also collected an additional dataset, which contains 5520 peptide segments, as the validation set. This dataset was ensured to have a similarity less than 0.75 with the training set through the CD-Hit-2d method, while maintaining a nearly 1:1 ratio of antibacterial peptides (n = 2,769) and non-antibacterial peptides (n = 2,751) in the dataset. To conducting high-throughput screening and experimental screening, this study randomly generated 250,000 peptides for subsequent screening.

The peptide sequences were embedded into 320-dimensional vectors, which corresponds to the internal hidden states of the ESM-2 model (specifically the esm2_t6_8 M_UR50D variant) (Cordoves-Delgado and García-Jacas, 2024). To determine the optimal embedding dimensionality, we compared the performance of ESM-2 models with 320 and 480 dimensions across various machine learning algorithms. Based on the results on the independent validation set (Supplementary Table S1), the 320-dimensional embedding was selected. This choice was made to balance computational efficiency and predictive accuracy while minimizing the risk of overfitting, especially considering the constraints of the training dataset size (Lin et al., 2023; Pan et al., 2024). The complete dataset was partitioned into training and testing subsets at an 80:20 ratio, followed by feature normalization to ensure uniform data distribution.

These ESM-2 embeddings were subsequently fed into a Transformer-based neural network designed to capture complex feature dependencies. This study employed a Transformer encoder to process 320-dimensional feature vectors derived from ESM-2. This encoder is configured with 10 layers and 8 attention heads, and the entire framework is implemented using the PyTorch library. To align with standard data formatting, the batch_first = True parameter is specified, while other settings for torch. nn.TransformerEncoder and torch. nn.TransformerEncoderLayer remain at their default values. The final binary classification is achieved by feeding the encoder’s output through a fully-connected linear layer followed by a Softmax activation function. Model training is conducted by minimizing a cross-entropy loss with the RAdam optimizer, which is configured with a learning rate of 1e^-5^ and a weight decay of 1e^-4^ (Pan et al., 2024). In parallel, several traditional machine learning models, including XGBoost, Random Forest, ExtraTrees, K-Nearest Neighbors, Decision Tree, Support Vector Classifier, and Artificial Neural Networks (multi-layer perceptron (MLP), and it was constructed using the scikit-learn library), were constructed for comparative analysis, with hyperparameters tuned to maximize performance.

### 2.3 Model evaluation

Following training, predictive outcomes were generated for the training, test, and independent validation datasets to comprehensively assess model effectiveness. Confusion matrices were computed using the confusion_matrix function from the sklearn. metrics package to quantify classification outcomes for antimicrobial and non-antimicrobial peptides. From these matrices, the values of true positives (TP), false positives (FP), false negatives (FN), and true negatives (TN) were derived. Based on Equations 1–7, multiple evaluation indices—namely Accuracy (ACC), Area Under the ROC Curve (AUC), Matthews Correlation Coefficient (MCC), Precision, F1-score, Specificity (Sp), and Sensitivity (Sn)—were calculated to provide an integrated evaluation of prediction accuracy, class balance, and discriminative capacity.

$$ACC=\frac{TP+TN}{TP+FP+FN+TN}$$ (1)

AUC=Area under the ROC curve (2)

$$MCC=\frac{TN\times TP-FN\times FP}{\sqrt{\left(TN+FP\right)\left(FN+TP\right)\left(TN+FN\right)\left(TP+FP\right)}}$$ (3)

$$Precision=\frac{TP}{TP+FP}$$ (4)

$$F1=\frac{2TP}{2TP+FP+FN}$$ (5)

$$Sp=\frac{\;TN}{TN+FP}$$ (6)

$$Sn=\frac{TP}{FN+TP}$$ (7)

### 2.4 Screening of AMPs

To efficiently mine candidate antimicrobial peptides (AMPs) from the randomly generated peptide pool, a multistage *in silico* screening workflow was established by integrating diverse bioinformatics tools. Initially, sequence clustering and redundancy reduction were conducted using CD-HIT with a similarity cutoff of 0.75, thereby minimizing repetitive sequences and improving downstream analytical robustness (Fu et al., 2012). Physicochemical properties of the nonredundant peptides, including net charge, secondary structure propensity, and the proportion of hydrophobic amino acids, were then calculated using the BioPython toolkit to assess whether the peptides were consistent with typical AMP characteristics (Cock et al., 2009). Thereafter, a series of predictive models were employed to comprehensively evaluate peptide bioactivity and biosafety. Toxicity potential was assessed via ToxinPred3 (Rathore et al., 2024), allergenicity risk was estimated using pLM4Alg (Du et al., 2024), and antimicrobial efficacy was predicted using the AMP classifier developed in this study. Moreover, hemolytic propensity was examined using the HemoPI2 algorithm (Rathore et al., 2025). Based on these integrated computational outputs, peptides exhibiting strong antimicrobial potential alongside favorable safety profiles were selected for experimental confirmation.

### 2.5 Solid-phase synthesis of AMPs

All shortlisted AMPs were produced using solid-phase peptide synthesis (SPPS) on Rink Amide MBHA resin following established protocols (Yang et al., 2025). The crude synthetic products were purified through reversed-phase high-performance liquid chromatography (RP-HPLC), achieving purities exceeding 95%. Molecular masses of the peptides were confirmed using an Agilent 6125B electrospray ionization mass spectrometer (Agilent, United States of America). Additional purity verification was carried out by RP-HPLC employing a PLRP-S 100 Å column (4.6 mm × 250 mm). The elution system consisted of water and acetonitrile supplemented with 0.1% trifluoroacetic acid (TFA), operated at a flow rate of 1.0 mL/min, with absorbance monitored at 220 nm.

### 2.6 Determination of MIC

Antibacterial activity of the synthesized peptides was evaluated by determining minimum inhibitory concentrations (MICs) using the two-fold broth microdilution technique. Peptides were prepared in sterile deionized water and serially diluted in 96-well microplates prior to the addition of bacterial suspensions, yielding final cell densities of 1 × 10^5^ to 5 × 10^5^ CFU mL^-1^. Following incubation at 37 °C for 20–24 h, bacterial growth was quantified by measuring optical density at 630 nm. Nisin Z served as the positive reference compound. Nisin Z was selected as the positive control due to its status as a widely recognized and clinically approved antimicrobial peptide. The research reports that lactic acid bacteria Z has a highly inhibitory effect on *Pseudomonas aeruginosa*, and the gel loaded with Nisin effectively treats the third-degree burn wounds of mice infected with *Pseudomonas aeruginosa* (Preet et al., 2021; Zina et al., 2023). Antimicrobial efficacy was calculated using the equation:

$$\text{Antimicrobial rate}\left(\%\right)=\frac{{OD}_{n}-{OD}_{p}}{{OD}_{n}-{OD}_{0}}\times 100$$

where OD_n_ represents the optical density of untreated bacterial cultures, OD_0_ corresponds to the broth blank, and OD_p_ denotes bacterial density following peptide exposure.

### 2.7 Scanning electron microscopy

Bacterial cultures suspended in PBS (OD_600_ = 0.2–0.5) were incubated with AMPs at 2× MIC and 4× MIC for 1 h at 37 °C with shaking at 150 rpm, while untreated cells served as controls. After treatment, cells were harvested, rinsed three times with PBS, and fixed overnight in 2.5% glutaraldehyde at 4 °C. Samples were subsequently washed and dehydrated sequentially in increasing ethanol concentrations (30%–100%), each step lasting 15 min. Dehydrated specimens were treated twice with isoamyl acetate for 20 min before undergoing critical point drying using a Leica EM CPD300 system. After gold sputter coating with a Leica EM ACE200, morphological alterations were visualized using a HITACHI SU8010 scanning electron microscope (Hitachi, Japan).

### 2.8 Molecular dynamics simulation of AMP–membrane interactions

To explore peptide–membrane interactions at the molecular level, all-atom molecular dynamics simulations were conducted according to the methodology described by Bennett et al. (2016). Peptide three-dimensional conformations were predicted via AlphaFold2 and parameterized using the Amber14sb force field. Model bacterial membranes were constructed using CHARMM-GUI Membrane Builder, incorporating DOPC and DOPG phospholipids to mimic bacterial lipid composition (Wu et al., 2014). Each bilayer leaflet consisted of 170 lipid molecules, with corresponding force field parameters derived from Amber14sb.

Peptides were initially positioned 5.0 nm above the bilayer surface prior to simulation. A total simulation duration of 400 ns was performed using GROMACS 21.6 (Abraham et al., 2015), with TIP3P employed as the solvent model. Systems were neutralized with 0.15 M NaCl and energy minimized through the steepest descent algorithm over 5000 steps, maintaining a force convergence threshold of 1000 kJ/mol/nm. Six equilibration stages of 500 ps each with positional restraints were conducted to stabilize membrane architecture. Thermodynamic control was achieved using the Nose–Hoover thermostat (Evans and Holian, 1985) and the Parrinello–Rahman barostat (Parrinello and Rahman, 1981) at 310.15 K and 1.0 bar. Hydrogen bonds were constrained via LINCS, long-range electrostatics were calculated using Particle-Mesh Ewald and van der Waals interactions were truncated at 1.2 nm. Post-simulation analyses were carried out using standard GROMACS tools.

### 2.9 Prokaryotic transcriptomic analysis

For the prokaryotic transcriptomic analysis, *Pseudomonas aeruginosa* (ATCC 27853) was cultured in LB medium at 37 °C with shaking at 150 rpm until the logarithmic growth phase was reached. The bacterial suspension was then divided into two groups: the untreated control group and the AP8-treated group. In the treatment group, AP8 was added to the culture at a final concentration of 4 MIC to induce significant transcriptional changes without causing immediate extensive cell lysis. The control group was treated with an equal volume of sterile phosphate-buffered saline (PBS). Both groups were incubated for an additional 1 h under the same conditions. After incubation, the cells were immediately harvested by centrifugation at 4,000 rpm for 10 min at 4 °C. The resulting cell pellets were rapidly frozen in liquid nitrogen and stored at −80 °C.

Total RNA was isolated from untreated controls (PA01–PA03) and AP8-exposed samples (PA11–PA13), followed by quality assessment prior to library preparation. Ribosomal RNA was removed, and strand-specific RNA sequencing libraries were generated in accordance with manufacturer protocols. Sequencing was performed on an Illumina platform to produce paired-end reads. Base calling was executed using Bcl2fastq (v2.17.1.14), and read quality was evaluated using FastQC (v0.10.1). Low-quality bases, adapter contaminants, ambiguous reads, and sequences shorter than 75 bp were eliminated using Cutadapt (v1.9.1), yielding high-confidence clean datasets. Processed reads were aligned to the *Pseudomonas aeruginosa* PAO1 reference genome (GCF_000006765.1) using Bowtie2 (v2.2.6). Alignment files were stored in BAM format and visualized with IGV. Transcript abundance was quantified using HTSeq (v0.6.1) and normalized as FPKM values.

Differential transcriptional profiling between control and AP8-treated samples was conducted using DESeq2 (v1.26.0) under a negative binomial statistical framework. Genes exhibiting |log_2_FC| ≥ 1 and FDR-adjusted p-values ≤ 0.05 were classified as significantly differentially expressed. Functional annotation and enrichment were performed using GO and KEGG databases. GO analysis employed the GOseq approach to correct for gene length bias, while KEGG pathway enrichment utilized hypergeometric testing, with Q-values < 0.05 indicating significance. Rich factor values were calculated as the proportion of DEGs mapped to each pathway relative to total annotated genes within that pathway.

### 2.10 Statistical analysis

All computational modeling and statistical evaluations were performed using Python 3.7, with hypothesis testing executed through the SciPy package. Experimental outcomes are expressed as mean ± standard deviation (SD). Statistical significance among groups was assessed using one-way ANOVA followed by LSD and Duncan *post hoc* comparisons, with p < 0.05 considered significant. Machine learning workflows were implemented using scikit-learn 1.7 and PyTorch, and data visualization was completed using Origin 8.5 software.

## 3 Results and analysis

### 3.1 Construction and evaluation of deep learning models

**FIGURE 1:**
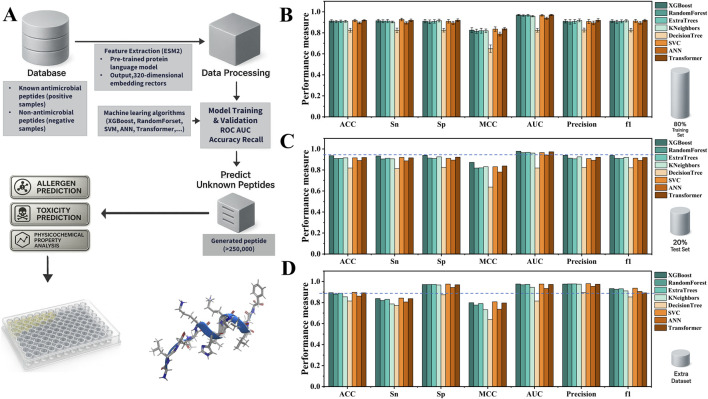
Workflow for deep learning–based antimicrobial peptide discovery. **(A)** Schematic overview of the proposed prediction framework integrating peptide sequence generation, feature extraction using ESM2, and classification by multiple machine learning algorithms. **(B–D)** Comparative performance of different machine learning models combined with ESM2 embeddings evaluated on the 80% training set and 20% test set **(B)**, the independent test set **(C)**, and an external validation dataset **(D)**, respectively.

As depicted in the computational screening pipeline in Figure 1A, datasets related to antibacterial, antifungal, antiviral, anti-mammalian cell, and anticancer activities were systematically compiled. Each antibacterial peptide dataset was encoded using the large language model ESM2, after which a predictive framework based on a Transformer network in combination with seven widely adopted machine learning methods was established. Model training incorporated cross-validation procedures, and predictive performance was assessed using an independent test dataset together with an external validation dataset. Upon completion of training, antibacterial activity was inferred for 25,000 artificially generated peptide sequences, followed by secondary screening of promising candidates through the integration of diverse bioinformatics strategies. The shortlisted peptides were subsequently subjected to experimental confirmation.

To comprehensively evaluate model performance, seven statistical indicators were employed: accuracy (ACC), area under the receiver operating characteristic curve (AUC), Matthews correlation coefficient (MCC), Precision, F1-score, specificity (Sp), and sensitivity (Sn). ACC reflects the proportion of correctly classified samples among all predictions and serves as an overall measure of accuracy. AUC quantifies the classifier’s discriminative capability across varying thresholds, with higher values indicating improved performance. MCC provides an integrated assessment of prediction quality by accounting for true positives (TP), true negatives (TN), false positives (FP), and false negatives (FN). Precision denotes the fraction of correctly predicted positive instances among all positive predictions, while the F1-score represents the harmonic mean of Precision and Recall (Sn), balancing these two aspects in binary classification. Sp measures the capacity to correctly recognize negative samples, whereas Sn evaluates the accurate identification of positive samples and is synonymous with Recall.

The predictive outcomes of eight machine learning algorithms combined with ESM2 on the 80% training dataset, 20% testing dataset, and an additional validation dataset are illustrated in Figures 1B–D, respectively. As shown in Figure 1B, all models achieved robust performance across multiple metrics in the training phase. Except for the Decision Tree approach, most algorithms yielded metric values exceeding 0.9. Comparable trends were observed in both the testing and external validation datasets, in agreement with previous studies (Guo et al., 2023; Zhang et al., 2024).

Collectively, these findings indicate that coupling ESM2-derived embeddings with diverse machine learning techniques enables highly accurate identification of antimicrobial peptides, underscoring the effectiveness of ESM2 representations in discriminating active peptides from inactive sequences. Among the evaluated models, XGBoost consistently delivered the most reliable and superior performance across all assessed indicators. Its precision reached 91%, 92%, and 97% in the training, testing, and validation datasets, respectively, and it ranked among the top performers for each of the seven evaluation metrics (Supplementary Tables S2, S3).

These outcomes align with a recent investigation utilizing four predictive algorithms, which likewise demonstrated the outstanding capability of XGBoost in identifying novel antimicrobial peptides targeting *Fusarium graminearum* (Ran et al., 2025). Taken together, the evidence supports XGBoost as a powerful and dependable classifier for distinguishing antimicrobial from non-antimicrobial peptides, thereby providing a solid methodological basis for high-precision peptide screening in future research.

To further verify the reliability of the model on unseen biological data, we performed an external validation using 124 experimentally validated AMPs sourced from recent publication (Guan et al., 2025; Huang et al., 2026; Li et al., 2026). The results showed that all 124 sequences were correctly identified as active antimicrobial peptides by our model. This high level of consistency with external experimental data demonstrates the robust generalization capability of the XGBoost framework. Detailed information regarding these sequences and their predicted scores is provided in Table S4.

### 3.2 Screening of antimicrobial peptides

**FIGURE 2:**
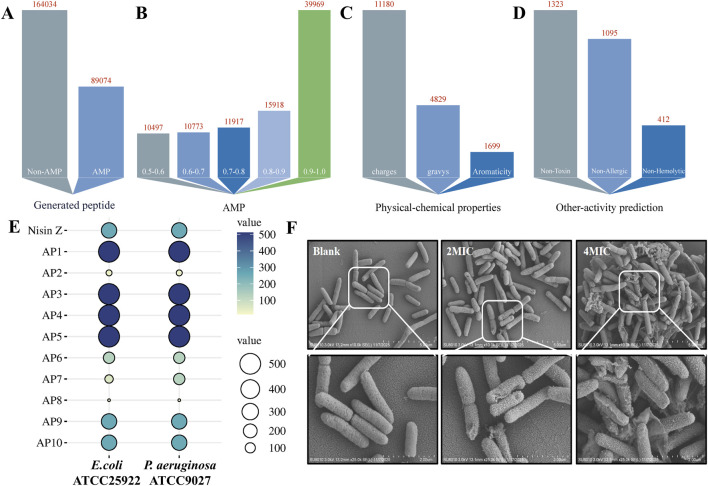
Screening and validation of antimicrobial peptides. **(A)** Antimicrobial activity prediction of more than 250,000 randomly generated peptide sequences using the established machine learning model. **(B)** Distribution of predicted activity scores and selection thresholds. **(C)** Physicochemical property-based filtering, including net charge between 2 and 5, GRAVY values between −0.5 and 0.5, and aromaticity ≥ 0.1. **(D)** Secondary antimicrobial activity prediction following physicochemical screening. **(E)** Minimum inhibitory concentration (MIC) values of synthesized peptides against *E. coli* ATCC25922 and *P. aeruginosa* ATCC9027. **(F)** SEM images of *P. aeruginosa* ATCC9027 cell membranes after treatment with AP8.

To further enhance the efficiency and reliability of antimicrobial peptide (AMP) screening, physicochemical properties and predicted biological activities were evaluated in addition to antimicrobial activity scores. As shown in Figure 2A, among the one million generated peptide sequences, 253,109 peptide segments were initially predicted to exhibit antimicrobial activity. After applying a prediction score threshold of 0.5, 89,074 peptide segments were classified as candidate AMPs, accounting for approximately 35.2% of all predicted antimicrobial sequences. Frequency distribution analysis of the prediction scores revealed that 39,969 peptides exhibited scores between 0.9 and 1.0, representing 44.9% of the predicted AMPs. Because higher prediction scores generally correlate with stronger antimicrobial potential, these high-confidence peptides were considered promising candidates for further screening.

AMPs are typically positively charged, with net charges commonly ranging from +2 to +5, which facilitates electrostatic interactions with negatively charged bacterial membranes. However, excessively high positive charge may increase cytotoxicity toward host cells (Zhou et al., 2019). In this study, physicochemical analysis indicated that 11,180 predicted AMPs possessed net charges within the +2 to +5 range, consistent with characteristic AMP properties and potentially associated with reduced toxicity. Hydrophobicity, as reflected by the grand average of hydropathicity (GRAVY) index, also plays a crucial role in membrane interaction and penetration. Peptides with GRAVY values greater than 0 tend to exhibit stronger hydrophobic character, promoting insertion into lipid bilayers and enhancing antimicrobial efficacy; however, excessive hydrophobicity can increase hemolytic activity (Gagat et al., 2024). In the present dataset, 4,829 predicted AMPs displayed GRAVY values between −0.5 and 0.5, indicating a balanced hydrophobic profile that may support effective membrane disruption while minimizing potential cytotoxic effects.

Furthermore, peptide toxicity, allergenicity, and hemolytic activity were incorporated as additional safety-related screening criteria (Krishnamoorthy et al., 2023). Among the predicted AMPs, 1,323 peptides were predicted to be non-toxic, 1,095 peptides were predicted to be non-allergenic, and 412 peptides were predicted to be non-hemolytic. Based on the combined criteria of antimicrobial activity prediction, physicochemical properties, and safety related features, ten peptides, AP1 to AP10 with sequences LNVLKRVKVEQRLNLF, GLTLKHLKKLIF, LVNKLKSVLAKY, IIKLILKHNKLAKIEY, VKKLRLEKVVLY, LSKNQKVLLRLF, RVSSKLRLLTLF, RKVRGPPRIWVIWRR, MAKYRIRI, and FRVVWGRRGL, were ultimately selected for subsequent experimental analyses.

### 3.3 Evaluation of antibacterial activity of candidate peptides

The ten candidate antimicrobial peptides were synthesized using solid-phase peptide synthesis, and their antibacterial activities against the pathogenic bacteria *Escherichia coli* ATCC25922 and *Pseudomonas aeruginosa* ATCC9027 were subsequently evaluated. The results are presented in Figure 2E. The well-characterized antimicrobial peptide Nisin Z was used as a positive control (Yuan et al., 2024). The minimum inhibitory concentration (MIC) values of Nisin Z against both *E. coli* ATCC25922 and *P. aeruginosa* ATCC9027 were 256 μg/mL. Among the ten candidate peptides, AP1, AP3, AP4, and AP5 exhibited relatively weak inhibitory effects against both bacterial strains. In contrast, the remaining six peptides demonstrated antibacterial activities comparable to or exceeding that of Nisin Z. Notably, AP2, AP6, AP7, and AP8 showed significantly stronger inhibitory activity than the positive control against both pathogens.

Specifically, AP2 and AP8 displayed MIC values of 32 μg/mL and 16 μg/mL, respectively, against both *E. coli* ATCC25922 and *P. aeruginosa* ATCC9027, indicating potent antibacterial activity. Overall, 60% of the screened peptides exhibited antibacterial performance equal to or superior to that of Nisin Z, demonstrating that the screening strategy employed in this study is highly effective and comparable to or superior to conventional antimicrobial peptide discovery approaches. Among these candidates, AP8 exhibited the strongest antibacterial activity and was therefore selected for subsequent mechanistic investigations.

Previous studies have shown that disruption of bacterial cell membrane integrity is one of the primary mechanisms underlying the antibacterial activity of antimicrobial peptides (Lou et al., 2024). Therefore, assessment of membrane integrity provides a direct indication of peptide-mediated antibacterial effects. In this study, scanning electron microscopy (SEM) was employed to examine the effects of AP8 at different concentrations on the cell membrane structure of *P. aeruginosa* ATCC9027 (Erviana et al., 2021). Three experimental groups were included: a Blank control group, a 2×MIC AP8 treatment group, and a 4×MIC AP8 treatment group. SEM images of the Blank group revealed intact bacterial cells with smooth surfaces, clear contours, and typical rod-shaped morphology with rounded ends, consistent with normal cellular structure (Kumaresan et al., 2016).

In contrast, pronounced morphological alterations were observed following AP8 treatment. After exposure to 2×MIC AP8, partial membrane rupture and cellular shrinkage were evident. At 4×MIC, most bacterial cells exhibited severe structural damage, characterized by extensive membrane collapse and pore formation. These observations indicate that AP8 exerts a strong, concentration-dependent antibacterial effect primarily through disruption of bacterial cell membrane integrity.

### 3.4 Molecular dynamics simulation analysis

**FIGURE 3:**
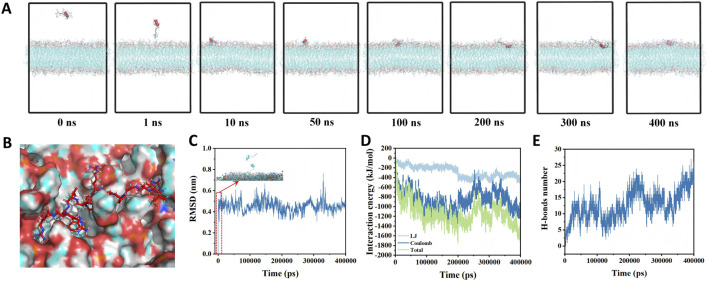
Molecular dynamics simulation of the interaction between antimicrobial peptide AP8 and the cell membrane. **(A)** Representative conformations of AP8 and the membrane at different simulation time points. **(B)** Conformation of AP8 embedded in the cell membrane. **(C)** The RMSD changes of the AP8 backbone during the molecular dynamics simulation process. **(D)** Interaction energy between AP8 and the cell membrane. **(E)** Number of hydrogen bonds formed between AP8 and the cell membrane.

To further elucidate the interaction between AP8 and the cell membrane at the molecular level, a 400 ns all-atom molecular dynamics (MD) simulation was performed using GROMACS, as illustrated in Figure 3. In the initial configuration, AP8 was positioned above the membrane without direct contact. Subsequently, an unrestrained 400 ns simulation was conducted to monitor the dynamic peptide–membrane interaction process. Representative simulation snapshots (Figure 3A) showed that AP8 began associating with the membrane at approximately 10 ns and progressively inserted into the lipid bilayer, with pronounced membrane penetration observed between 100 and 400 ns? Conformational analysis further indicated that AP8 adopted a stable, tightly bound configuration within the membrane and formed extensive interactions with surrounding lipid molecules, consistent with strong peptide–membrane binding behavior (Yang et al., 2025).

To ensure that the simulation reaches a balanced state, we calculated the RMSD results of AP8. The results showed that when AP8 binds to the cell membrane (≈10 ns), its conformation remains in a relatively stable state, indicating that the simulation has reached a balanced state (Figure 3C). Analysis of the interaction energy between AP8 and the membrane revealed a fluctuating decrease during the 0–100 ns interval, followed by a pronounced increase around 100 ns and a gradual decrease during the 100–400 ns interval (Figure 3D). The Lennard–Jones (LJ) term represents van der Waals interactions, including short-range repulsive and intermediate-range attractive forces, whereas the Coulomb term corresponds to electrostatic interactions. The sum of these components reflects the total non-bonded interaction energy between AP8 and the membrane. The observed energy variation suggests that the initial decrease corresponds to the approach of AP8 toward the membrane surface, while between 50 and 100 ns? AP8 gradually inserts into and binds to the lipid bilayer. A lower total interaction energy indicates a more stable peptide–membrane association (Zou et al., 2018).

Hydrogen bond analysis (Figure 3E) further demonstrated that AP8 formed stable interactions with the membrane during the 50–400 ns period, maintaining approximately 15–20 hydrogen bonds. These hydrogen bonds contributed significantly to the sustained peptide–membrane association and structural stability of the complex (Kim et al., 2022). Since the cell membrane is in a flowing state during the simulation, this causes fluctuations in its binding energy and hydrogen bonds with AP8. In addition, molecular dynamics simulations were used to calculate the mean square displacement (MSD) of molecules in the MEMB (Blank) and MEMB (Model) systems (Supplementary Figure S1). In both systems, MSD increased approximately linearly with time, indicating normal diffusion behavior within the selected time scale. Linear fitting of the MSD–time curves yielded diffusion coefficients of 0.0205 × 10^-5^ cm^2^/s for the MEMB (Blank) system and 0.0173 × 10^-5^ cm^2^/s for the MEMB (Model) system. Compared with the Blank membrane, molecular diffusion in the Model membrane was markedly reduced, suggesting that AP8 binding imposed structural constraints on membrane dynamics and decreased overall molecular mobility (Lai and Kaznessis, 2018). Taken together, these MD simulation results provide molecular-level insights into the antibacterial mechanism of AP8, demonstrating that AP8 stably associates with and disrupts membrane structure, thereby contributing to its potent antibacterial activity observed *in vitro*.

### 3.5 Transcriptomic analysis

**FIGURE 4:**
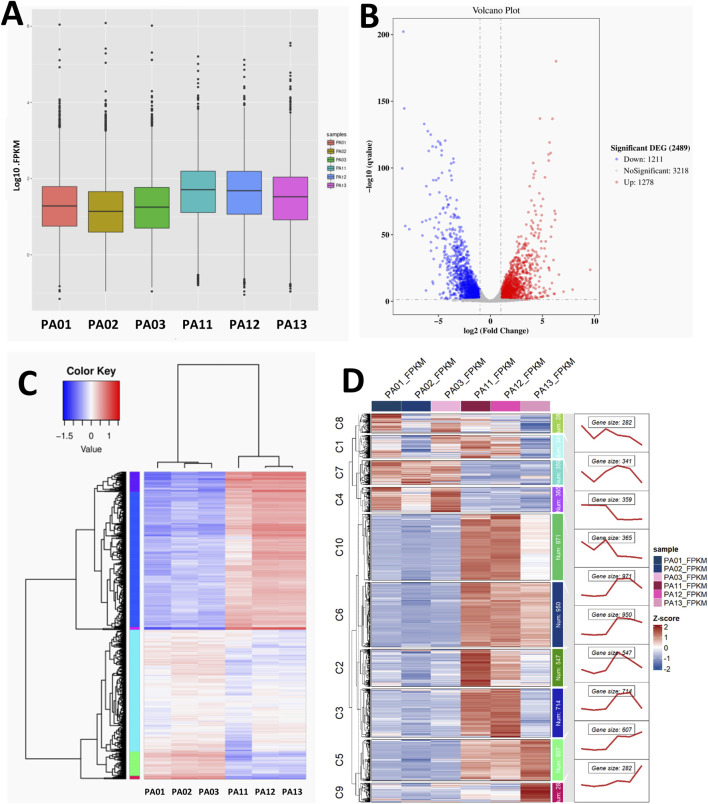
Differential gene expression between untreated controls (PA01–PA03) and AP8 exposed samples (PA11–PA13). **(A)** Boxplots showing the distribution of log_10_ (FPKM) expression values across samples. Expression was quantified using HTSeq (v0.6.1) and normalized by the FPKM method (Fragments Per Kilobase of transcript per Million mapped reads). The x-axis indicates sample identifiers, and the y-axis represents log_10_-transformed FPKM values. Each box summarizes the maximum, upper quartile, median, lower quartile, and minimum, enabling assessment of expression uniformity across biological replicates. **(B)** Volcano plot of differentially expressed genes. Significance thresholds were set at |log_2_ (fold change)| ≥ 1 and adjusted p-value (false discovery rate, q-value) ≤ 0.05. Upregulated genes following AP8 treatment are shown in red, downregulated genes in blue, and non-significant genes in gray. **(C)** Hierarchical clustering heatmap of differentially expressed genes. Red and blue indicate relatively high and low expression levels, respectively, with distinct colored regions representing separate expression clusters. **(D)** Heatmap of Z-score-normalized FPKM expression patterns. Genes were grouped into ten temporal expression modules (C0–C9), with cluster sizes indicated. Line graphs below show average expression trajectories across samples, where color transitions from blue (low expression) to red (high expression).

To comprehensively explore the molecular basis of AP8-mediated antibacterial activity, RNA sequencing was conducted on untreated bacterial samples (PA01, PA02, PA03) alongside AP8-exposed groups (PA11–PA13) (Younas et al., 2025). As illustrated in Figure 4A, gene expression profiles displayed uniform distribution patterns across all samples, reflecting high data reliability and strong intergroup comparability. Subsequent differential expression profiling revealed substantial transcriptomic shifts following AP8 exposure (Figure 4B). Using stringent thresholds (|log_2_FC| ≥ 1 and padj ≤ 0.05), 2,489 genes exhibited significant expression changes, comprising 1,278 genes with elevated expression and 1,211 genes showing reduced expression, indicating extensive regulatory remodeling induced by AP8. Unsupervised hierarchical clustering of these differentially expressed genes (Figure 4C) distinctly separated control and treated groups while maintaining strong consistency among biological replicates, demonstrating a stable transcriptional response to AP8 treatment. Moreover, Z-score clustering partitioned the DEGs into ten expression modules (Figure 4D), among which clusters C10, C6, C2, C3, and C5 displayed strong induction after AP8 exposure, whereas clusters C8, C1, C4, and C7 were prominently suppressed. Overall, these changes indicate that the addition of AP8 has affected the metabolic functions of the bacteria.

**FIGURE 5:**
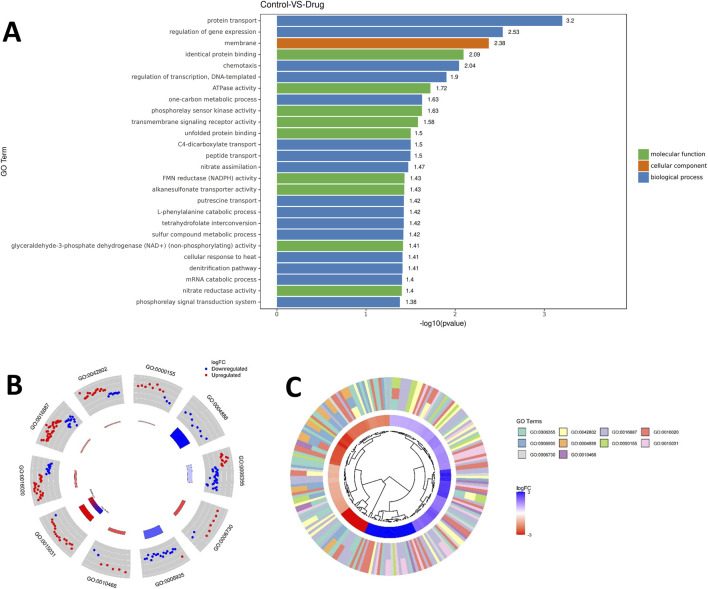
Gene Ontology (GO) functional enrichment analysis of differentially expressed genes (DEGs) following AP8 treatment. **(A)** Bar plot of GO enrichment results comparing the control and AP8-treated groups. The y-axis represents significantly enriched GO terms, and the x-axis indicates −log_10_ (P value), with higher values reflecting greater enrichment significance. Different colors denote GO functional categories: Biological Process (BP, blue), Molecular Function (MF, red), and Cellular Component (CC, green). **(B)** GO chord diagram illustrating the relationships between DEGs and significantly enriched GO terms. Red represents upregulated genes, blue represents downregulated genes, and chords connect genes to their associated functional terms, highlighting gene–function associations. **(C)** GO cluster diagram showing the distribution and expression patterns of DEGs across different GO functional clusters. Inner circle colors indicate gene expression changes (log_2_FC), while outer circle colors represent distinct GO functional categories.

To clarify the biological significance of transcriptional alterations triggered by AP8, Gene Ontology enrichment analysis was conducted on the set of differentially expressed genes, revealing widespread functional perturbations across numerous categories (Figure 5). Within the Biological Process domain, enriched terms were primarily associated with protein translocation, control of gene expression, bacterial chemotaxis, one-carbon metabolism, sulfur compound metabolism, and denitrification-related processes (Figure 5A; Supplementary Table S5), all of which are integral to microbial metabolic regulation, environmental responsiveness, and cellular survival, suggesting that AP8 disrupts essential adaptive and metabolic functions (Klaysubun et al., 2026).

In the Molecular Function category, significant enrichment was observed for ATP hydrolysis activity, receptor-mediated transmembrane signaling, phosphorelay sensor kinases, and unfolded protein binding, indicating interference with energy utilization, signal transduction mechanisms, and protein quality control systems. Enrichment within the Cellular Component classification predominantly involved membrane-associated structures. The GO chord visualization further demonstrated that numerous enriched terms encompassed both transcriptionally upregulated and downregulated genes (Figure 5B), reflecting the presence of complex regulatory shifts rather than unidirectional responses. Moreover, GO cluster analysis revealed coordinated expression groupings across functional modules, highlighting synchronized regulatory alterations among interconnected biological pathways (Figure 5C). Overall, these findings suggest that AP8 may exert its antibacterial effect by influencing the stress responses and membrane-related processes of bacteria (Klaysubun et al., 2026).

**FIGURE 6:**
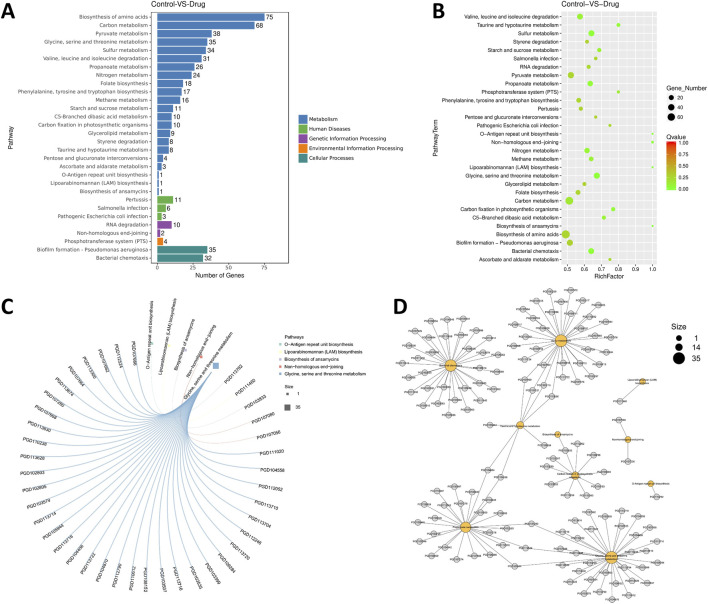
KEGG pathway enrichment analysis of differentially expressed genes (DEGs) following AP8 treatment. **(A)** KEGG annotation classification bar chart of DEGs. The y-axis indicates KEGG pathway categories, and the x-axis shows the number of DEGs annotated to each pathway. Different colors represent major functional classes, including metabolism, genetic information processing, environmental information processing, cellular processes, and human diseases. **(B)** KEGG enrichment scatters plot of DEGs. The y-axis represents KEGG pathway names, and the x-axis indicates the Rich factor (ratio of DEGs to total genes annotated within each pathway). Bubble size corresponds to the number of DEGs, and bubble color denotes enrichment significance (Q value). **(C)** KEGG chord diagram illustrating the associations between significantly enriched pathways and DEGs. Different colors represent pathway categories, and connecting lines indicate genes involved in corresponding pathways. **(D)** KEGG cluster network diagram showing interaction relationships between DEGs and enriched KEGG pathways. Node size reflects the degree of connectivity, indicating the regulatory centrality of key pathways and associated genes.

Further KEGG pathway enrichment analysis was conducted on the differentially expressed genes (DEGs) between the control and AP8-treated groups to identify the biological pathways affected by AP8 exposure (Figure 6). KEGG annotation classification revealed that DEGs were predominantly distributed among metabolism-related pathways, followed by pathways associated with environmental information processing, genetic information processing, and cellular processes (Figure 6A). These findings indicate that AP8 treatment exerts a pronounced impact on bacterial basal metabolic functions (Ren et al., 2022). The KEGG enrichment scatter plot demonstrated that numerous metabolic pathways were significantly enriched with DEGs, including amino acid biosynthesis, carbon metabolism, pyruvate metabolism, sulfur metabolism, branched-chain amino acid degradation, and glycine, serine, and threonine metabolism (Figure 6B) (Ye et al., 2018). Several of these pathways exhibited high Rich factors and low Q values, indicating strong and statistically significant regulation in response to AP8 treatment. In addition to metabolic pathways, functional pathways related to bacterial chemotaxis, biofilm formation, and the phosphotransferase system (PTS) were also significantly enriched, suggesting that AP8 disrupts bacterial environmental sensing, nutrient transport, and adaptive behaviors.

The KEGG chord diagram further illustrated extensive associations between significantly enriched pathways and numerous DEGs (Figure 6C). Notably, 35 enriched pathways were linked to glycine, serine, and threonine metabolism, highlighting complex transcriptional regulation of amino acid metabolic networks following AP8 exposure. KEGG cluster network analysis revealed that metabolism-related pathways occupied central positions within the interaction network and exhibited high connectivity with multiple functional pathways (Figure 6D). For instance, carbohydrate metabolism, taurine metabolism, pyruvate metabolism, and glycine, serine, and threonine metabolism were interconnected through shared DEGs, indicating coordinated regulation across metabolic systems.

## 4 Conclusion

In this study, an efficient antimicrobial peptide prediction model combining machine learning and traditional machine learning algorithms was successfully developed. By screening many randomly generated peptides, ten antimicrobial peptides with potential activity were identified. The antibacterial activities of these peptides were validated through solid-phase synthesis and MIC assays, among which AP8 exhibited strong activity against *Pseudomonas aeruginosa*. SEM analysis demonstrated that AP8 could significantly disrupt the integrity of the bacterial cell membrane. Molecular dynamics simulations further revealed the interacting mechanism between AP8 and the cell membrane, indicating that AP8 exerts its antibacterial effect by inserting into and disrupting membrane structures. Transcriptomic analysis revealed a global metabolic dysfunction because of AP8 treatment, which further accelerated bacterial death following initial membrane damage. These findings provide new theoretical support for the rational design of antimicrobial peptides and offer valuable insights into the development of antibiotic alternatives.

However, the limitation of this study lies in the scarcity of current datasets for the annotation of AMPs subtypes, as well as the high sequence variability and structural flexibility. This makes it difficult to evaluate the model performance in a hierarchical manner like for traditional proteins. In the future, we will continue to build dedicated annotation datasets for AMPs subtypes, conduct hierarchical performance analysis of the models, and develop high-precision prediction models for AMPs tailored to different subtypes.

## Data Availability

The original contributions presented in the study are included in the article, further inquiries can be directed to the corresponding authors.

## References

[B1] Abraham M. J. Murtola T. Schulz R. Páll S. Smith J. C. Hess B. (2015). GROMACS: high performance molecular simulations through multi-level parallelism from laptops to supercomputers. SoftwareX 1-2, 19–25. 10.1016/j.softx.2015.06.001

[B2] Bennett W. F. D. Hong C. K. Wang Y. Tieleman D. P. (2016). Antimicrobial peptide simulations and the influence of force field on the free energy for pore formation in lipid bilayers. J. Chem. Theory Comput. 12 (9), 4524–4533. 10.1021/acs.jctc.6b00265 27529120

[B3] Bournez C. Riool M. de Boer L. Cordfunke R. A. de Best L. van Leeuwen R. (2023). CalcAMP: a new machine learning model for the accurate prediction of antimicrobial activity of peptides. Antibiotics 12 (4), 725. 10.3390/antibiotics12040725 37107088 PMC10135148

[B4] Chen S. Qi H. Zhu X. Liu T. Fan Y. Su Q. (2024). Screening and identification of antimicrobial peptides from the gut microbiome of cockroach *Blattella germanica* . Microbiome 12 (1), 272. 10.1186/s40168-024-01985-9 39709489 PMC11663339

[B5] Cock P. J. A. Antao T. Chang J. T. Chapman B. A. Cox C. J. Dalke A. (2009). Biopython: freely available python tools for computational molecular biology and bioinformatics. Bioinformatics 25 (11), 1422–1423. 10.1093/bioinformatics/btp163 19304878 PMC2682512

[B6] Cordoves-Delgado G. García-Jacas C. R. (2024). Predicting antimicrobial peptides using ESMFold-Predicted structures and ESM-2-Based amino acid features with graph deep learning. J. Chem. Inf. Model. 64 (10), 4310–4321. 10.1021/acs.jcim.3c02061 38739853

[B7] Du Z. Comer J. Li Y. (2023). Bioinformatics approaches to discovering food-derived bioactive peptides: reviews and perspectives. TrAC Trends Anal. Chem. 162, 117051. 10.1016/j.trac.2023.117051

[B8] Du Z. Xu Y. Liu C. Li Y. (2024). pLM4Alg: protein language model-based predictors for allergenic proteins and peptides. J. Agric. Food Chem. 72 (1), 752–760. 10.1021/acs.jafc.3c07143 38113537

[B9] Erviana R. Saengkun Y. Rungsa P. Jangpromma N. Tippayawat P. Klaynongsruang S. (2021). Novel antimicrobial peptides from a cecropin-like region of Heteroscorpine-1 from Heterometrus laoticus venom with membrane disruption activity. Molecules 26 (19), 5872. 10.3390/molecules26195872 34641415 PMC8512776

[B10] Evans D. J. Holian B. L. (1985). The nose–hoover thermostat. J. Chem. Phys. 83 (8), 4069–4074. 10.1063/1.449071

[B11] Fu L. Niu B. Zhu Z. Wu S. Li W. (2012). CD-HIT: accelerated for clustering the next-generation sequencing data. Bioinformatics 28 (23), 3150–3152. 10.1093/bioinformatics/bts565 23060610 PMC3516142

[B12] Gagat P. Ostrówka M. Duda-Madej A. Mackiewicz P. (2024). Enhancing antimicrobial peptide activity through modifications of charge, hydrophobicity, and structure. Int. J. Mol. Sci. 25 (19), 10821. 10.3390/ijms251910821 39409150 PMC11476776

[B13] Guan C. Torres M. D. T. Li S. de la Fuente-Nunez C. (2025). Computational exploration of global venoms for antimicrobial discovery with venomics artificial intelligence. Nat. Commun. 16 (1), 6446. 10.1038/s41467-025-60051-6 40645962 PMC12254355

[B14] Guo T. Pan F. Cui Z. Yang Z. Chen Q. Zhao L. (2023). FAPD: an astringency threshold and astringency type prediction database for flavonoid compounds based on machine learning. J. Agric. Food Chem. 71 (9), 4172–4183. 10.1021/acs.jafc.2c08822 36825752

[B15] Huang J. Zhang W. Wang A. Jiang Y. Lai Y. Xu Y. (2026). Discovery of antimicrobial peptides targeting Acinetobacter baumannii via a pre-trained and fine-tuned few-shot learning-based pipeline. Nat. Commun. 17 (1), 2475. 10.1038/s41467-026-69306-2 41654506 PMC12992665

[B16] Igídio C. E. D. Brito C. B. Bezerra R. d. O. Oliveira S. N. Teixeira C. F. Amorim-Santos B. M. d. (2025). Indole-acetic acid impairs *Pseudomonas aeruginosa* virulence and alters lung infection in mice. MicrobiologyOpen 14 (6), e70185. 10.1002/mbo3.70185 41384457 PMC12699321

[B17] Inda-Díaz J. S. Lund D. Parras-Moltó M. Johnning A. Bengtsson-Palme J. Kristiansson E. (2023). Latent antibiotic resistance genes are abundant, diverse, and Mobile in human, animal, and environmental microbiomes. Microbiome 11 (1), 44. 10.1186/s40168-023-01479-0 36882798 PMC9993715

[B18] Jhong J.-H. Chi Y.-H. Li W.-C. Lin T.-H. Huang K.-Y. Lee T.-Y. (2019). dbAMP: an integrated resource for exploring antimicrobial peptides with functional activities and physicochemical properties on transcriptome and proteome data. Nucleic Acids Res. 47 (D1), D285–D297. 10.1093/nar/gky1030 30380085 PMC6323920

[B19] Joseph S. Karnik S. Nilawe P. Jayaraman V. K. Idicula-Thomas S. (2012). ClassAMP: a prediction tool for classification of antimicrobial peptides. IEEE/ACM Trans. Comput. Biol. Bioinforma. 9 (5), 1535–1538. 10.1109/TCBB.2012.89 22732690

[B20] Ju R. Li Y. Sui D. Xu F.-J. (2025). Polyaminoglycoside nanosystem expressing antimicrobial peptides for multistage chronic wound management. J. Control. Release 382, 113657. 10.1016/j.jconrel.2025.113657 40122239

[B21] Khan A. U. Maryam L. Zarrilli R. (2017). Structure, genetics and worldwide spread of New Delhi Metallo-β-lactamase (NDM): a threat to public health. BMC Microbiol. 17 (1), 101. 10.1186/s12866-017-1012-8 28449650 PMC5408368

[B22] Kim H. Yoo Y. D. Lee G. Y. (2022). Identification of bacterial membrane selectivity of Romo1-Derived antimicrobial peptide AMPR-22 via molecular dynamics. Int. J. Mol. Sci. 23 (13), 7404. 10.3390/ijms23137404 35806412 PMC9266825

[B23] Klaysubun C. Kompramool S. Dechathai T. Chaichana N. Suwannasin S. Singkhamanan K. (2026). Transcriptomic insights into the antimicrobial effect of Pediococcus pentosaceus on multidrug-resistant Pseudomonas aeruginosa. Infect. Genet. Evol. 138, 105879. 10.1016/j.meegid.2026.105879 41539587

[B24] Kok M. Maton L. van der Peet M. Hankemeier T. van Hasselt J. G. C. (2022). Unraveling antimicrobial resistance using metabolomics. Drug Discov. Today 27 (6), 1774–1783. 10.1016/j.drudis.2022.03.015 35341988

[B25] Krishnamoorthy R. Adhikari P. Anaikutti P. (2023). Design, synthesis, and characterization of non-hemolytic antimicrobial peptides related to human cathelicidin LL-37 [10.1039/D3RA02473C]. RSC Adv. 13 (23), 15594–15605. 10.1039/D3RA02473C 37228679 PMC10204126

[B26] Kumaresan V. Bhatt P. Arockiaraj J. (2016). Membrane disruption antimicrobial mechanism of Channa striatus lysozyme-derived antimicrobial peptides (AMP). Fish and Shellfish Immunol. 53, 74–75. 10.1016/j.fsi.2016.03.091

[B27] Lai P.-K. Kaznessis Y. N. (2018). Insights into membrane translocation of protegrin antimicrobial peptides by multistep molecular dynamics simulations. ACS Omega 3 (6), 6056–6065. 10.1021/acsomega.8b00483 29978143 PMC6026836

[B28] Li X. Gong H. Wang Y. Zhao Y. Li L. Bao P. (2026). *De novo* Multi-Mechanism Antimicrobial Peptide Design via Multimodal Deep Learning. Adv. Sci. n/a(n/a) 13, e15835. 10.1002/advs.202515835 PMC13185819 41801219

[B29] Lin Z. Akin H. Rao R. Hie B. Zhu Z. Lu W. (2023). Evolutionary-scale prediction of atomic-level protein structure with a language model. Science 379 (6637), 1123–1130. 10.1126/science.ade2574 36927031

[B30] Liu X. Li J. Zhang Z. He Y. Wang M. Zhao Y. (2024). Acetylation of xenogeneic silencer H-NS regulates biofilm development through the nitrogen homeostasis regulator in shewanella. Nucleic Acids Res. 52 (6), 2886–2903. 10.1093/nar/gkad1219 38142446 PMC11014242

[B31] Lopatkin A. J. Bening S. C. Manson A. L. Stokes J. M. Kohanski M. A. Badran A. H. (2021). Clinically relevant mutations in core metabolic genes confer antibiotic resistance. Science 371 (6531), eaba0862. 10.1126/science.aba0862 33602825 PMC8285040

[B32] Lou T. Zhuang X. Chang J. Gao Y. Bai X. (2024). Effect of surface-immobilized states of antimicrobial peptides on their ability to disrupt bacterial cell membrane structure. J. Funct. Biomaterials 15 (11), 315. 10.3390/jfb15110315 PMC11595214 39590519

[B33] Ma Y. Guo Z. Xia B. Zhang Y. Liu X. Yu Y. (2022). Identification of antimicrobial peptides from the human gut microbiome using deep learning. Nat. Biotechnol. 40 (6), 921–931. 10.1038/s41587-022-01226-0 35241840

[B34] Mahlapuu M. Håkansson J. Ringstad L. Björn C. (2016). Antimicrobial peptides: an emerging category of therapeutic agents. Front. Cell. Infect. Microbiol. 6, 00194. 10.3389/fcimb.2016.00194 28083516 PMC5186781

[B35] Manyi-Loh C. Mamphweli S. Meyer E. Okoh A. (2018). Antibiotic use in agriculture and its consequential resistance in environmental sources: potential public health implications. Molecules 23 (4), 795. 10.3390/molecules23040795 29601469 PMC6017557

[B36] Pan F. Liu D. Tuersuntuoheti T. Xing H. Zhu Z. Fang Y. (2024). Mining anti-hypertensive peptides in animal food through deep learning: a case study of gastrointestinal digestive products of royal jelly. Food Sci. Animal Prod. 2 (1), 9240053. 10.26599/FSAP.2024.9240053

[B37] Parrinello M. Rahman A. (1981). Polymorphic transitions in single crystals: a new molecular dynamics method. J. Appl. Phys. 52, 7182–7190. 10.1063/1.328693

[B38] Preet S. Kaur J. Raza K. (2021). Nisin loaded carbopol gel against Pseudomonas aeruginosa infected third-degree burns: a therapeutic intervention. Wound Repair Regen. 29 (5), 711–724. 10.1111/wrr.12909 33721379

[B39] Ran Y. Li S. Wang Y.-J. Liang J.-H. Jiang W. Yu M.-J. (2025). AI-Accelerated identification of novel antimicrobial peptides for inhibiting Fusarium graminearum. J. Agric. Food Chem. 73 (28), 17471–17482. 10.1021/acs.jafc.5c03429 40598766

[B40] Rather M. A. Gupta K. Mandal M. (2021). Microbial biofilm: formation, architecture, antibiotic resistance, and control strategies. Braz. J. Microbiol. 52 (4), 1701–1718. 10.1007/s42770-021-00624-x 34558029 PMC8578483

[B41] Rathore A. S. Choudhury S. Arora A. Tijare P. Raghava G. P. S. (2024). ToxinPred 3.0: an improved method for predicting the toxicity of peptides. Comput. Biol. Med. 179, 108926. 10.1016/j.compbiomed.2024.108926 39038391

[B42] Rathore A. S. Kumar N. Choudhury S. Mehta N. K. Raghava G. P. S. (2025). Prediction of hemolytic peptides and their hemolytic concentration. Commun. Biol. 8 (1), 176. 10.1038/s42003-025-07615-w 39905233 PMC11794569

[B43] Ren X. Liu J. Ndandala C. B. Li X. Guo Y. Li G. (2022). Physiological effects and transcriptomic analysis of sbGnRH on the liver in pompano (Trachinotus ovatus). Front. Endocrinol. 13, 869021. 10.3389/fendo.2022.869021 PMC9108241 35586618

[B44] Rivero-Pino F. Millan-Linares M. C. Montserrat-de-la-Paz S. (2023). Strengths and limitations of *in silico* tools to assess physicochemical properties, bioactivity, and bioavailability of food-derived peptides. Trends Food Sci. and Technol. 138, 433–440. 10.1016/j.tifs.2023.06.023

[B45] Rutherford V. Yom K. Ozer E. A. Pura O. Hughes A. Murphy K. R. (2018). Environmental reservoirs for exoS+ and exoU+ strains of Pseudomonas aeruginosa. Environ. Microbiol. Rep. 10 (4), 485–492. 10.1111/1758-2229.12653 29687624 PMC6108916

[B46] Shan X. Yin B. Liao X. Xiao B. He J. Li C. (2024). Exploration and characterization of antimicrobial peptides from shrimp litopenaeus vannamei by A genomic and transcriptomic approach. Mar. Biotechnol. 26 (5), 975–990. 10.1007/s10126-024-10358-0 39138702

[B47] Sharma R. Shrivastava S. Kumar Singh S. Kumar A. Saxena S. Kumar Singh R. (2021). AniAMPpred: artificial intelligence guided discovery of novel antimicrobial peptides in animal kingdom. Briefings Bioinforma. 22 (6), bbab242. 10.1093/bib/bbab242 34259329

[B48] Verhoeve V. I. Brammer J. A. Driscoll T. P. Kambouris A. R. Rasko D. A. Cross A. S. (2022). Genome sequencing of Pseudomonas aeruginosa strain M2 illuminates traits of an opportunistic pathogen of burn wounds. G3 Genes Genomes Genetics 12 (5), jkac073. 10.1093/g3journal/jkac073 35348684 PMC9073672

[B49] Vos T. Lim S. S. Abbafati C. Abbas K. M. Abbasi M. Abbasifard M. (2020). Global burden of 369 diseases and injuries in 204 countries and territories, 1990–2019: a systematic analysis for the global burden of disease study 2019. Lancet 396 (10258), 1204–1222. 10.1016/S0140-6736(20)30925-9 33069326 PMC7567026

[B50] Wu E. L. Cheng X. Jo S. Rui H. Song K. C. Dávila-Contreras E. M. (2014). CHARMM-GUI membrane builder toward realistic biological membrane simulations. J. Comput. Chem. 35 (27), 1997–2004. 10.1002/jcc.23702 25130509 PMC4165794

[B51] Xu J. Li F. Li C. Guo X. Landersdorfer C. Shen H.-H. (2023). iAMPCN: a deep-learning approach for identifying antimicrobial peptides and their functional activities. Briefings Bioinforma. 24 (4), bbad240. 10.1093/bib/bbad240 PMC10359087 37369638

[B52] Yang Q. E. Ma X. Li M. Zhao M. Zeng L. He M. (2024). Evolution of triclosan resistance modulates bacterial permissiveness to multidrug resistance plasmids and phages. Nat. Commun. 15 (1), 3654. 10.1038/s41467-024-48006-9 38688912 PMC11061290

[B53] Yang H. Pan F. Wang L. Duan B. Gao J. Tian W. (2025). Hydrophobic group modification for constructing self-assembling antimicrobial peptide derivatives with superior antimicrobial performance. Chem. Eng. J. 512, 162645. 10.1016/j.cej.2025.162645

[B54] Yang D. Li Y. Li C. Zhang Q. Huang J. Li X. (2026). Biochemical-knowledge-driven machine learning pipeline for generating potent antimicrobial peptides. Briefings Bioinforma. 27 (2), bbag115. 10.1093/bib/bbag115 PMC12998437 41849223

[B55] Ye J.-z. Lin X.-m. Cheng Z.-x. Su Y.-b. Li W.-x. Ali F.-m. (2018). Identification and efficacy of glycine, serine and threonine metabolism in potentiating kanamycin-mediated killing of Edwardsiella piscicida. J. Proteomics 183, 34–44. 10.1016/j.jprot.2018.05.006 29753025

[B56] Younas M. Wang C. Hassan M. F. Li W. Zheng Z. Bin Y. (2025). Comprehensive transcriptomic profiling of citrus australasica unveils antimicrobial peptides and immune pathways for huanglongbing tolerance. J. Agric. Food Chem. 73 (27), 16847–16859. 10.1021/acs.jafc.4c11910 40497554

[B57] Yu G. Baeder D. Y. Regoes R. R. Rolff J. (2018). Predicting drug resistance evolution: insights from antimicrobial peptides and antibiotics. Proc. R. Soc. B Biol. Sci. 285 (1874), 20172687. 10.1098/rspb.2017.2687 29540517 PMC5879628

[B58] Yuan F. Zhang Z. Fang Z. (2023). An effective CNN and transformer complementary network for medical image segmentation. Pattern Recognit. 136, 109228. 10.1016/j.patcog.2022.109228

[B59] Yuan L. Wu S. Tian K. Wang S. Wu H. Qiao J. (2024). Nisin-relevant antimicrobial peptides: synthesis strategies and applications [10.1039/D3FO05619H]. Food and Funct. 15 (19), 9662–9677. 10.1039/D3FO05619H 39246095

[B60] Zhang Y. Zhu Y. Bao X. Dai Z. Shen Q. Wang L. (2024). Mining bovine milk proteins for DPP-4 inhibitory peptides using machine learning and virtual proteolysis. Research 7, 0391. 10.34133/research.0391 38887277 PMC11182572

[B61] Zhou J. Chen L. Liu Y. Shen T. Zhang C. Liu Z. (2019). Antimicrobial peptide PMAP-37 analogs: increasing the positive charge to enhance the antibacterial activity of PMAP-37. J. Peptide Sci. 25 (12), e3220. 10.1002/psc.3220 31858653

[B62] Zina R. Cunha E. Serrano I. Silva E. Tavares L. Oliveira M. (2023). Nisin Z potential for the control of diabetic foot infections promoted by Pseudomonas aeruginosa persisters. Antibiotics 12 (5), 794. 10.3390/antibiotics12050794 37237697 PMC10215260

[B63] Zou R. Zhu X. Tu Y. Wu J. Landry M. P. (2018). Activity of antimicrobial peptide aggregates decreases with increased cell membrane embedding free energy cost. Biochemistry 57 (18), 2606–2610. 10.1021/acs.biochem.8b00052 29638118 PMC10493161

