# Supplementary material for: Machine learning–driven discovery of antimicrobial peptides against Pseudomonas aeruginosa
Source: Front Pharmacol. 2026 Jun 24;17:1837055. doi: 10.3389/fphar.2026.1837055 (PMC13341293; doi:10.3389/fphar.2026.1837055)
Supplement: Supplementary file 1 [file Supplementaryfile1.docx]

Table S1. Performance comparison of different algorithms combined with 320-dimensional and 480-dimensional embedding vectors on the independent validation set.

| **Algorithm** | **320 Vector** | **480 Vector** |
| --- | --- | --- |
| XGBoost | 0.92 | 0.91 |
| Random Forest (RF) | 0.91 | 0.91 |
| ExtraTrees | 0.91 | 0.89 |
| KNeighbors | 0.92 | 0.90 |
| Decision Tree | 0.82 | 0.85 |
| SVC | 0.92 | 0.87 |
| ANN | 0.89 | 0.88 |
| Transformer | 0.92 | 0.89 |

Table S2. Performance comparison of 8 ML models on the testing set (20%, n = 2002).

| **Model** | **Metric** | **Non-AMP (991)** | **AMP (1011)** | **Accuracy** |
| --- | --- | --- | --- | --- |
| XGBoost | Precision | 0.92 | 0.92 | / |
|  | Recall | 0.92 | 0.92 | / |
|  | F1-score | 0.92 | 0.92 | 0.92 |
| Random Forest (RF) | Precision | 0.91 | 0.91 | / |
|  | Recall | 0.91 | 0.91 | / |
|  | F1-score | 0.91 | 0.91 | 0.91 |
| ExtraTrees | Precision | 0.91 | 0.91 | / |
|  | Recall | 0.91 | 0.91 | / |
|  | F1-score | 0.91 | 0.91 | 0.91 |
| KNeighbors | Precision | 0.91 | 0.93 | / |
|  | Recall | 0.93 | 0.91 | / |
|  | F1-score | 0.92 | 0.92 | 0.92 |
| Decision Tree | Precision | 0.81 | 0.82 | / |
|  | Recall | 0.82 | 0.81 | / |
|  | F1-score | 0.82 | 0.82 | 0.82 |
| SVC | Precision | 0.92 | 0.91 | / |
|  | Recall | 0.91 | 0.92 | / |
|  | F1-score | 0.91 | 0.92 | 0.92 |
| ANN | Precision | 0.89 | 0.89 | / |
|  | Recall | 0.89 | 0.89 | / |
|  | F1-score | 0.89 | 0.89 | 0.89 |
| Transformer | Precision | 0.92 | 0.92 | / |
|  | Recall | 0.92 | 0.92 | / |
|  | F1-score | 0.92 | 0.92 | 0.92 |

Table S3. Performance comparison of 8 ML models on the external set (n = 5520).

| Model | Metric | Non-AMP (2769) | AMP (2751) | Accuracy |
| --- | --- | --- | --- | --- |
| XGBoost | Precision | 0.84 | 0.97 | / |
|  | Recall | 0.98 | 0.81 | / |
|  | F1-score | 0.9 | 0.88 | 0.89 |
| Random Forest (RF) | Precision | 0.83 | 0.97 | / |
|  | Recall | 0.97 | 0.79 | / |
|  | F1-score | 0.89 | 0.87 | 0.88 |
| ExtraTrees | Precision | 0.83 | 0.97 | / |
|  | Recall | 0.98 | 0.8 | / |
|  | F1-score | 0.9 | 0.88 | 0.89 |
| KNeighbors | Precision | 0.79 | 0.97 | / |
|  | Recall | 0.98 | 0.73 | / |
|  | F1-score | 0.87 | 0.84 | 0.86 |
| Decision Tree | Precision | 0.81 | 0.82 | / |
|  | Recall | 0.82 | 0.81 | / |
|  | F1-score | 0.82 | 0.82 | 0.82 |
| SVC | Precision | 0.92 | 0.91 | / |
|  | Recall | 0.91 | 0.92 | / |
|  | F1-score | 0.91 | 0.92 | 0.92 |
| ANN | Precision | 0.89 | 0.89 | / |
|  | Recall | 0.89 | 0.89 | / |
|  | F1-score | 0.89 | 0.89 | 0.89 |
| Transformer | Precision | 0.92 | 0.92 | / |
|  | Recall | 0.92 | 0.92 | / |
|  | F1-score | 0.92 | 0.92 | 0.92 |

Table S4. Prediction results for 124 antimicrobial peptides reported in recent publications.

| **Sequence** | **Inactive** | **Active** | DOI |
| --- | --- | --- | --- |
| LWWWRR | 0.000517905 | 0.999482095 | https://doi.org/10.1038/s41467-026-69306-2 |
| IWRWWR | 0.000164628 | 0.999835372 | https://doi.org/10.1038/s41467-026-69306-2 |
| IWWWRR | 0.000227749 | 0.999772251 | https://doi.org/10.1038/s41467-026-69306-2 |
| WLWWRR | 0.000141501 | 0.999858499 | https://doi.org/10.1038/s41467-026-69306-2 |
| KLWRWW | 0.000150323 | 0.999849677 | https://doi.org/10.1038/s41467-026-69306-2 |
| KWIKLW | 9.03606E-05 | 0.999909639 | https://doi.org/10.1038/s41467-026-69306-2 |
| KWFRLW | 0.000998318 | 0.999001682 | https://doi.org/10.1038/s41467-026-69306-2 |
| KWAKLW | 2.36034E-05 | 0.999976397 | https://doi.org/10.1038/s41467-026-69306-2 |
| RWIRWI | 0.000137925 | 0.999862075 | https://doi.org/10.1038/s41467-026-69306-2 |
| RWIRWW | 3.94583E-05 | 0.999960542 | https://doi.org/10.1038/s41467-026-69306-2 |
| RWWRWI | 0.000206411 | 0.999793589 | https://doi.org/10.1038/s41467-026-69306-2 |
| KWIRIW | 6.59227E-05 | 0.999934077 | https://doi.org/10.1038/s41467-026-69306-2 |
| RWIRWV | 3.76701E-05 | 0.99996233 | https://doi.org/10.1038/s41467-026-69306-2 |
| RIWRWW | 9.47714E-05 | 0.999905229 | https://doi.org/10.1038/s41467-026-69306-2 |
| RWIRVW | 3.8743E-05 | 0.999961257 | https://doi.org/10.1038/s41467-026-69306-2 |
| KWIRWW | 6.2108E-05 | 0.999937892 | https://doi.org/10.1038/s41467-026-69306-2 |
| WFRWIK | 3.00407E-05 | 0.999969959 | https://doi.org/10.1038/s41467-026-69306-2 |
| RWFRIW | 0.000114083 | 0.999885917 | https://doi.org/10.1038/s41467-026-69306-2 |
| RWIHFF | 0.003669798 | 0.996330202 | https://doi.org/10.1038/s41467-026-69306-2 |
| WIRIWH | 8.63075E-05 | 0.999913692 | https://doi.org/10.1038/s41467-026-69306-2 |
| RWIHFW | 0.000404894 | 0.999595106 | https://doi.org/10.1038/s41467-026-69306-2 |
| RSWCLW | 0.010448515 | 0.989551485 | https://doi.org/10.1038/s41467-026-69306-2 |
| KSWCLW | 0.003624618 | 0.996375382 | https://doi.org/10.1038/s41467-026-69306-2 |
| RWIRWF | 0.000360608 | 0.999639392 | https://doi.org/10.1038/s41467-026-69306-2 |
| RWFRWI | 0.000252903 | 0.999747097 | https://doi.org/10.1038/s41467-026-69306-2 |
| RWIRIW | 7.06911E-05 | 0.999929309 | https://doi.org/10.1038/s41467-026-69306-2 |
| KWFRIW | 0.000156403 | 0.999843597 | https://doi.org/10.1038/s41467-026-69306-2 |
| RIFRWW | 0.000390232 | 0.999609768 | https://doi.org/10.1038/s41467-026-69306-2 |
| KWWKWI | 1.93119E-05 | 0.999980688 | https://doi.org/10.1038/s41467-026-69306-2 |
| RWWKIW | 7.7486E-05 | 0.999922514 | https://doi.org/10.1038/s41467-026-69306-2 |
| KWWKLI | 9.42945E-05 | 0.999905705 | https://doi.org/10.1038/s41467-026-69306-2 |
| RRWWRIW | 0.000192106 | 0.999807894 | https://doi.org/10.1038/s41467-026-69306-2 |
| RRWIKLW | 5.75781E-05 | 0.999942422 | https://doi.org/10.1038/s41467-026-69306-2 |
| RWIRWIH | 0.000116348 | 0.999883652 | https://doi.org/10.1038/s41467-026-69306-2 |
| RRWFRWI | 0.000123382 | 0.999876618 | https://doi.org/10.1038/s41467-026-69306-2 |
| RRWIRIW | 5.05447E-05 | 0.999949455 | https://doi.org/10.1038/s41467-026-69306-2 |
| KRWFRIW | 7.96318E-05 | 0.999920368 | https://doi.org/10.1038/s41467-026-69306-2 |
| KRWIRIW | 3.0756E-05 | 0.999969244 | https://doi.org/10.1038/s41467-026-69306-2 |
| RRWIRWFR | 4.98295E-05 | 0.999950171 | https://doi.org/10.1038/s41467-026-69306-2 |
| RRWIRWWK | 2.22921E-05 | 0.999977708 | https://doi.org/10.1038/s41467-026-69306-2 |
| RRWFRWIR | 3.94583E-05 | 0.999960542 | https://doi.org/10.1038/s41467-026-69306-2 |
| RRWWKLIR | 4.26769E-05 | 0.999957323 | https://doi.org/10.1038/s41467-026-69306-2 |
| KRWIRWLT | 0.000106335 | 0.999893665 | https://doi.org/10.1038/s41467-026-69306-2 |
| RRWWRILK | 9.97782E-05 | 0.999900222 | https://doi.org/10.1038/s41467-026-69306-2 |
| RRWFRWIT | 6.12736E-05 | 0.999938726 | https://doi.org/10.1038/s41467-026-69306-2 |
| RRWIRWIT | 0.000151515 | 0.999848485 | https://doi.org/10.1038/s41467-026-69306-2 |
| RRWIRWIH | 5.76973E-05 | 0.999942303 | https://doi.org/10.1038/s41467-026-69306-2 |
| RRWIKLIK | 2.39611E-05 | 0.999976039 | https://doi.org/10.1038/s41467-026-69306-2 |
| KRWIKWLT | 1.77622E-05 | 0.999982238 | https://doi.org/10.1038/s41467-026-69306-2 |
| RRWWTVV | 0.000133872 | 0.999866128 | https://doi.org/10.1038/s41467-026-69306-2 |
| RGWIRWW | 2.15769E-05 | 0.999978423 | https://doi.org/10.1038/s41467-026-69306-2 |
| RRWWTLV | 0.000120163 | 0.999879837 | https://doi.org/10.1038/s41467-026-69306-2 |
| RRWWTVI | 0.000160694 | 0.999839306 | https://doi.org/10.1038/s41467-026-69306-2 |
| RKAIRWW | 0.000259697 | 0.999740303 | https://doi.org/10.1038/s41467-026-69306-2 |
| RRWWTLI | 0.000537932 | 0.999462068 | https://doi.org/10.1038/s41467-026-69306-2 |
| RAVRIWW | 8.57115E-05 | 0.999914289 | https://doi.org/10.1038/s41467-026-69306-2 |
| RAVIRWW | 0.000172019 | 0.999827981 | https://doi.org/10.1038/s41467-026-69306-2 |
| KWKIKWPSRWFRKL | 2.98023E-05 | 0.999970198 | https://doi.org/10.1002/advs.202515835 |
| KWKIKWPSNWFTKK | 4.27961E-05 | 0.999957204 | https://doi.org/10.1002/advs.202515835 |
| KVMIKWPSYWFTMR | 0.001774251 | 0.998225749 | https://doi.org/10.1002/advs.202515835 |
| KYKIKWPYRWFRKL | 0.000201046 | 0.999798954 | https://doi.org/10.1002/advs.202515835 |
| KWMIKTPSHWFTMR | 0.01875335 | 0.98124665 | https://doi.org/10.1002/advs.202515835 |
| KWMIKWPSHWFTVP | 0.000988245 | 0.999011755 | https://doi.org/10.1002/advs.202515835 |
| KHMIKWPRNWFTVL | 0.000285208 | 0.999714792 | https://doi.org/10.1002/advs.202515835 |
| KWMIIWPSKWFTVL | 0.001300454 | 0.998699546 | https://doi.org/10.1002/advs.202515835 |
| KWMIKWKTKWFVML | 0.002311945 | 0.997688055 | https://doi.org/10.1002/advs.202515835 |
| KWMIKWPLKWIIMKL | 0.000189841 | 0.999810159 | https://doi.org/10.1002/advs.202515835 |
| KRMIKWRVKWFTML | 6.40154E-05 | 0.999935985 | https://doi.org/10.1002/advs.202515835 |
| KWKIKWPVRWFRKL | 6.67572E-06 | 0.999993324 | https://doi.org/10.1002/advs.202515835 |
| KWKIKWPSNWFTKL | 0.000142932 | 0.999857068 | https://doi.org/10.1002/advs.202515835 |
| KWKIKWPSRWFTKL | 0.000173867 | 0.999826133 | https://doi.org/10.1002/advs.202515835 |
| KWKIKRPVKWFTML | 7.27177E-05 | 0.999927282 | https://doi.org/10.1002/advs.202515835 |
| KWKIKQWVRWFRKL | 2.02656E-06 | 0.999997973 | https://doi.org/10.1002/advs.202515835 |
| KRKIKWKVRWFTYL | 8.41618E-05 | 0.999915838 | https://doi.org/10.1002/advs.202515835 |
| KRKIKWWVRWITKL | 4.29153E-06 | 0.999995708 | https://doi.org/10.1002/advs.202515835 |
| KWKIKWRVRWFKLL | 1.83582E-05 | 0.999981642 | https://doi.org/10.1002/advs.202515835 |
| KWKIKWHVRWFRVL | 1.01328E-05 | 0.999989867 | https://doi.org/10.1002/advs.202515835 |
| KWKIKWVYRWFRKL | 5.24521E-06 | 0.999994755 | https://doi.org/10.1002/advs.202515835 |
| KWMIKWPSKWFRMY | 0.000105023 | 0.999894977 | https://doi.org/10.1002/advs.202515835 |
| KWKIKWKSNWFTML | 0.000901461 | 0.999098539 | https://doi.org/10.1002/advs.202515835 |
| KWMIKWPSKWCSMK | 0.001315653 | 0.998684347 | https://doi.org/10.1002/advs.202515835 |
| KWMIKWRVKWRTML | 0.001276076 | 0.998723924 | https://doi.org/10.1002/advs.202515835 |
| KWMIKWPSHWFVMR | 0.002236485 | 0.997763515 | https://doi.org/10.1002/advs.202515835 |
| KWMIKWVSKWRTKL | 0.000141978 | 0.999858022 | https://doi.org/10.1002/advs.202515835 |
| KWMIKWVSKWKRML | 0.000322938 | 0.999677062 | https://doi.org/10.1002/advs.202515835 |
| KWKIKWRVRWFRIL | 4.52995E-06 | 0.99999547 | https://doi.org/10.1002/advs.202515835 |
| KWMHKWPSKWFVMI | 0.000822842 | 0.999177158 | https://doi.org/10.1002/advs.202515835 |
| LLSGILKSLL | 0.000700712 | 0.999299288 | https://doi.org/10.1038/s41467-025-60051-6 |
| NWKKVLGKIIK | 2.59876E-05 | 0.999974012 | https://doi.org/10.1038/s41467-025-60051-6 |
| KIYRSWIGLKI | 0.000255883 | 0.999744117 | https://doi.org/10.1038/s41467-025-60051-6 |
| LTKWLGKLGVIL | 1.39475E-05 | 0.999986053 | https://doi.org/10.1038/s41467-025-60051-6 |
| ILGLLKGISALL | 0.003414273 | 0.996585727 | https://doi.org/10.1038/s41467-025-60051-6 |
| KIWRSKLVKRLR | 1.26362E-05 | 0.999987364 | https://doi.org/10.1038/s41467-025-60051-6 |
| KFITHFWIGLRI | 0.000908375 | 0.999091625 | https://doi.org/10.1038/s41467-025-60051-6 |
| FLGALLKIGAKL | 0.000275314 | 0.999724686 | https://doi.org/10.1038/s41467-025-60051-6 |
| KVSKFILWIKRI | 1.12057E-05 | 0.999988794 | https://doi.org/10.1038/s41467-025-60051-6 |
| WLGSALKIGAKLL | 0.000517368 | 0.999482632 | https://doi.org/10.1038/s41467-025-60051-6 |
| GKWLISSLVAKHL | 0.006432116 | 0.993567884 | https://doi.org/10.1038/s41467-025-60051-6 |
| KLKKLRKWIYRIV | 5.09024E-05 | 0.999949098 | https://doi.org/10.1038/s41467-025-60051-6 |
| KWLGKLGVILSHL | 0.000345826 | 0.999654174 | https://doi.org/10.1038/s41467-025-60051-6 |
| RRVKRFKKFFMKL | 2.57492E-05 | 0.999974251 | https://doi.org/10.1038/s41467-025-60051-6 |
| SRFILWIKRIMRL | 2.81334E-05 | 0.999971867 | https://doi.org/10.1038/s41467-025-60051-6 |
| FWSFLVKAASKIL | 4.54187E-05 | 0.999954581 | https://doi.org/10.1038/s41467-025-60051-6 |
| VWLSALKFIGKHL | 0.000954807 | 0.999045193 | https://doi.org/10.1038/s41467-025-60051-6 |
| FILWIKRIMRLKL | 0.000100136 | 0.999899864 | https://doi.org/10.1038/s41467-025-60051-6 |
| FWGAVWKILSKVL | 8.58307E-06 | 0.999991417 | https://doi.org/10.1038/s41467-025-60051-6 |
| IKFLAAWIFLKKF | 0.000314474 | 0.999685526 | https://doi.org/10.1038/s41467-025-60051-6 |
| RGNNKIALRFLLK | 0.000331998 | 0.999668002 | https://doi.org/10.1038/s41467-025-60051-6 |
| IFGSLFSLGSKLL | 0.000457048 | 0.999542952 | https://doi.org/10.1038/s41467-025-60051-6 |
| KLKDILGKIKVIL | 0.000194848 | 0.999805152 | https://doi.org/10.1038/s41467-025-60051-6 |
| IWGTALKWGVKLL | 9.50098E-05 | 0.99990499 | https://doi.org/10.1038/s41467-025-60051-6 |
| LFGFLIKLIPSLF | 0.000142336 | 0.999857664 | https://doi.org/10.1038/s41467-025-60051-6 |
| LLRFLLGRGGLLL | 9.83477E-05 | 0.999901652 | https://doi.org/10.1038/s41467-025-60051-6 |
| VFGKILRSIAKVF | 4.22001E-05 | 0.9999578 | https://doi.org/10.1038/s41467-025-60051-6 |
| VLGKVGGLIKKLL | 0.005429089 | 0.994570911 | https://doi.org/10.1038/s41467-025-60051-6 |
| LPLILGKLVKGLL | 0.000121474 | 0.999878526 | https://doi.org/10.1038/s41467-025-60051-6 |
| ILVSRFKISALTL | 0.016205966 | 0.983794034 | https://doi.org/10.1038/s41467-025-60051-6 |
| KGSKMLFSIYRKL | 0.000492573 | 0.999507427 | https://doi.org/10.1038/s41467-025-60051-6 |
| KFIVLLGALLALL | 0.002482295 | 0.997517705 | https://doi.org/10.1038/s41467-025-60051-6 |
| FLVNILTTLLTKL | 0.052274764 | 0.947725236 | https://doi.org/10.1038/s41467-025-60051-6 |
| SFKRLKGFAKKLW | 1.57356E-05 | 0.999984264 | https://doi.org/10.1038/s41467-025-60051-6 |
| RFGRLIRLIVKIA | 9.41753E-06 | 0.999990582 | https://doi.org/10.1038/s41467-025-60051-6 |
| KILLKLKEYLEKL | 0.001039386 | 0.998960614 | https://doi.org/10.1038/s41467-025-60051-6 |
| AGKLLRSLKKLKL | 2.52724E-05 | 0.999974728 | https://doi.org/10.1038/s41467-025-60051-6 |

Table S5. Results of GO functional enrichment analysis of differentially expressed genes after treatment with antibacterial peptide AP8.

| category | term | ontology | numDEInCat | numInCat | over_represented_pvalue | over_represented_FDR | GeneNumber(Up) | GeneNumber(Down) |
| --- | --- | --- | --- | --- | --- | --- | --- | --- |
| GO:0015031 | protein transport | BP | 17 | 21 | 0.00063 | 1 | [16](file:///C:\Users\亚西\Desktop\BQ-PF20251106-原核转录2G-XJCD-6-潘飞老师-项目交付\Result\09_GO\Control-VS-Drug\Control-VS-Drug.GO_0015031_Ups.xlsx) | [1](file:///C:\Users\亚西\Desktop\BQ-PF20251106-原核转录2G-XJCD-6-潘飞老师-项目交付\Result\09_GO\Control-VS-Drug\Control-VS-Drug.GO_0015031_Down.xlsx) |
| GO:0010468 | regulation of gene expression | BP | 7 | 7 | 0.002932 | 1 | [5](file:///C:\Users\亚西\Desktop\BQ-PF20251106-原核转录2G-XJCD-6-潘飞老师-项目交付\Result\09_GO\Control-VS-Drug\Control-VS-Drug.GO_0010468_Ups.xlsx) | [2](file:///C:\Users\亚西\Desktop\BQ-PF20251106-原核转录2G-XJCD-6-潘飞老师-项目交付\Result\09_GO\Control-VS-Drug\Control-VS-Drug.GO_0010468_Down.xlsx) |
| GO:0016020 | membrane | CC | 35 | 56 | 0.004207 | 1 | [23](file:///C:\Users\亚西\Desktop\BQ-PF20251106-原核转录2G-XJCD-6-潘飞老师-项目交付\Result\09_GO\Control-VS-Drug\Control-VS-Drug.GO_0016020_Ups.xlsx) | [12](file:///C:\Users\亚西\Desktop\BQ-PF20251106-原核转录2G-XJCD-6-潘飞老师-项目交付\Result\09_GO\Control-VS-Drug\Control-VS-Drug.GO_0016020_Down.xlsx) |
| GO:0042802 | identical protein binding | MF | 23 | 35 | 0.008075 | 1 | [15](file:///C:\Users\亚西\Desktop\BQ-PF20251106-原核转录2G-XJCD-6-潘飞老师-项目交付\Result\09_GO\Control-VS-Drug\Control-VS-Drug.GO_0042802_Ups.xlsx) | [8](file:///C:\Users\亚西\Desktop\BQ-PF20251106-原核转录2G-XJCD-6-潘飞老师-项目交付\Result\09_GO\Control-VS-Drug\Control-VS-Drug.GO_0042802_Down.xlsx) |
| GO:0006935 | chemotaxis | BP | 18 | 26 | 0.009067 | 1 | [1](file:///C:\Users\亚西\Desktop\BQ-PF20251106-原核转录2G-XJCD-6-潘飞老师-项目交付\Result\09_GO\Control-VS-Drug\Control-VS-Drug.GO_0006935_Ups.xlsx) | [17](file:///C:\Users\亚西\Desktop\BQ-PF20251106-原核转录2G-XJCD-6-潘飞老师-项目交付\Result\09_GO\Control-VS-Drug\Control-VS-Drug.GO_0006935_Down.xlsx) |
| GO:0006355 | regulation of transcription, DNA-templated | BP | 39 | 67 | 0.012567 | 1 | [13](file:///C:\Users\亚西\Desktop\BQ-PF20251106-原核转录2G-XJCD-6-潘飞老师-项目交付\Result\09_GO\Control-VS-Drug\Control-VS-Drug.GO_0006355_Ups.xlsx) | [26](file:///C:\Users\亚西\Desktop\BQ-PF20251106-原核转录2G-XJCD-6-潘飞老师-项目交付\Result\09_GO\Control-VS-Drug\Control-VS-Drug.GO_0006355_Down.xlsx) |
| GO:0016887 | ATPase activity | MF | 52 | 94 | 0.018985 | 1 | [36](file:///C:\Users\亚西\Desktop\BQ-PF20251106-原核转录2G-XJCD-6-潘飞老师-项目交付\Result\09_GO\Control-VS-Drug\Control-VS-Drug.GO_0016887_Ups.xlsx) | [16](file:///C:\Users\亚西\Desktop\BQ-PF20251106-原核转录2G-XJCD-6-潘飞老师-项目交付\Result\09_GO\Control-VS-Drug\Control-VS-Drug.GO_0016887_Down.xlsx) |
| GO:0006730 | one-carbon metabolic process | BP | 8 | 10 | 0.023581 | 1 | [6](file:///C:\Users\亚西\Desktop\BQ-PF20251106-原核转录2G-XJCD-6-潘飞老师-项目交付\Result\09_GO\Control-VS-Drug\Control-VS-Drug.GO_0006730_Ups.xlsx) | [2](file:///C:\Users\亚西\Desktop\BQ-PF20251106-原核转录2G-XJCD-6-潘飞老师-项目交付\Result\09_GO\Control-VS-Drug\Control-VS-Drug.GO_0006730_Down.xlsx) |
| GO:0000155 | phosphorelay sensor kinase activity | MF | 11 | 15 | 0.023625 | 1 | [8](file:///C:\Users\亚西\Desktop\BQ-PF20251106-原核转录2G-XJCD-6-潘飞老师-项目交付\Result\09_GO\Control-VS-Drug\Control-VS-Drug.GO_0000155_Ups.xlsx) | [3](file:///C:\Users\亚西\Desktop\BQ-PF20251106-原核转录2G-XJCD-6-潘飞老师-项目交付\Result\09_GO\Control-VS-Drug\Control-VS-Drug.GO_0000155_Down.xlsx) |
| GO:0004888 | transmembrane signaling receptor activity | MF | 8 | 10 | 0.026239 | 1 | [0](file:///C:\Users\亚西\Desktop\BQ-PF20251106-原核转录2G-XJCD-6-潘飞老师-项目交付\Result\09_GO\Control-VS-Drug\Control-VS-Drug.GO_0004888_Ups.xlsx) | [8](file:///C:\Users\亚西\Desktop\BQ-PF20251106-原核转录2G-XJCD-6-潘飞老师-项目交付\Result\09_GO\Control-VS-Drug\Control-VS-Drug.GO_0004888_Down.xlsx) |
| GO:0051082 | unfolded protein binding | MF | 9 | 12 | 0.03159 | 1 | [3](file:///C:\Users\亚西\Desktop\BQ-PF20251106-原核转录2G-XJCD-6-潘飞老师-项目交付\Result\09_GO\Control-VS-Drug\Control-VS-Drug.GO_0051082_Ups.xlsx) | [6](file:///C:\Users\亚西\Desktop\BQ-PF20251106-原核转录2G-XJCD-6-潘飞老师-项目交付\Result\09_GO\Control-VS-Drug\Control-VS-Drug.GO_0051082_Down.xlsx) |
| GO:0015740 | C4-dicarboxylate transport | BP | 6 | 7 | 0.031599 | 1 | [3](file:///C:\Users\亚西\Desktop\BQ-PF20251106-原核转录2G-XJCD-6-潘飞老师-项目交付\Result\09_GO\Control-VS-Drug\Control-VS-Drug.GO_0015740_Ups.xlsx) | [3](file:///C:\Users\亚西\Desktop\BQ-PF20251106-原核转录2G-XJCD-6-潘飞老师-项目交付\Result\09_GO\Control-VS-Drug\Control-VS-Drug.GO_0015740_Down.xlsx) |
| GO:0015833 | peptide transport | BP | 9 | 12 | 0.031761 | 1 | [9](file:///C:\Users\亚西\Desktop\BQ-PF20251106-原核转录2G-XJCD-6-潘飞老师-项目交付\Result\09_GO\Control-VS-Drug\Control-VS-Drug.GO_0015833_Ups.xlsx) | [0](file:///C:\Users\亚西\Desktop\BQ-PF20251106-原核转录2G-XJCD-6-潘飞老师-项目交付\Result\09_GO\Control-VS-Drug\Control-VS-Drug.GO_0015833_Down.xlsx) |
| GO:0042128 | nitrate assimilation | BP | 6 | 7 | 0.033654 | 1 | [3](file:///C:\Users\亚西\Desktop\BQ-PF20251106-原核转录2G-XJCD-6-潘飞老师-项目交付\Result\09_GO\Control-VS-Drug\Control-VS-Drug.GO_0042128_Ups.xlsx) | [3](file:///C:\Users\亚西\Desktop\BQ-PF20251106-原核转录2G-XJCD-6-潘飞老师-项目交付\Result\09_GO\Control-VS-Drug\Control-VS-Drug.GO_0042128_Down.xlsx) |
| GO:0052873 | FMN reductase (NADPH) activity | MF | 4 | 4 | 0.037096 | 1 | [2](file:///C:\Users\亚西\Desktop\BQ-PF20251106-原核转录2G-XJCD-6-潘飞老师-项目交付\Result\09_GO\Control-VS-Drug\Control-VS-Drug.GO_0052873_Ups.xlsx) | [2](file:///C:\Users\亚西\Desktop\BQ-PF20251106-原核转录2G-XJCD-6-潘飞老师-项目交付\Result\09_GO\Control-VS-Drug\Control-VS-Drug.GO_0052873_Down.xlsx) |
| GO:0042959 | alkanesulfonate transporter activity | MF | 4 | 4 | 0.037264 | 1 | [4](file:///C:\Users\亚西\Desktop\BQ-PF20251106-原核转录2G-XJCD-6-潘飞老师-项目交付\Result\09_GO\Control-VS-Drug\Control-VS-Drug.GO_0042959_Ups.xlsx) | [0](file:///C:\Users\亚西\Desktop\BQ-PF20251106-原核转录2G-XJCD-6-潘飞老师-项目交付\Result\09_GO\Control-VS-Drug\Control-VS-Drug.GO_0042959_Down.xlsx) |
| GO:0015847 | putrescine transport | BP | 4 | 4 | 0.037726 | 1 | [4](file:///C:\Users\亚西\Desktop\BQ-PF20251106-原核转录2G-XJCD-6-潘飞老师-项目交付\Result\09_GO\Control-VS-Drug\Control-VS-Drug.GO_0015847_Ups.xlsx) | [0](file:///C:\Users\亚西\Desktop\BQ-PF20251106-原核转录2G-XJCD-6-潘飞老师-项目交付\Result\09_GO\Control-VS-Drug\Control-VS-Drug.GO_0015847_Down.xlsx) |
| GO:0006559 | L-phenylalanine catabolic process | BP | 4 | 4 | 0.037775 | 1 | [0](file:///C:\Users\亚西\Desktop\BQ-PF20251106-原核转录2G-XJCD-6-潘飞老师-项目交付\Result\09_GO\Control-VS-Drug\Control-VS-Drug.GO_0006559_Ups.xlsx) | [4](file:///C:\Users\亚西\Desktop\BQ-PF20251106-原核转录2G-XJCD-6-潘飞老师-项目交付\Result\09_GO\Control-VS-Drug\Control-VS-Drug.GO_0006559_Down.xlsx) |
| GO:0035999 | tetrahydrofolate interconversion | BP | 4 | 4 | 0.037823 | 1 | [3](file:///C:\Users\亚西\Desktop\BQ-PF20251106-原核转录2G-XJCD-6-潘飞老师-项目交付\Result\09_GO\Control-VS-Drug\Control-VS-Drug.GO_0035999_Ups.xlsx) | [1](file:///C:\Users\亚西\Desktop\BQ-PF20251106-原核转录2G-XJCD-6-潘飞老师-项目交付\Result\09_GO\Control-VS-Drug\Control-VS-Drug.GO_0035999_Down.xlsx) |
| GO:0006790 | sulfur compound metabolic process | BP | 4 | 4 | 0.038168 | 1 | [4](file:///C:\Users\亚西\Desktop\BQ-PF20251106-原核转录2G-XJCD-6-潘飞老师-项目交付\Result\09_GO\Control-VS-Drug\Control-VS-Drug.GO_0006790_Ups.xlsx) | [0](file:///C:\Users\亚西\Desktop\BQ-PF20251106-原核转录2G-XJCD-6-潘飞老师-项目交付\Result\09_GO\Control-VS-Drug\Control-VS-Drug.GO_0006790_Down.xlsx) |
| GO:0043878 | glyceraldehyde-3-phosphate dehydrogenase (NAD+) (non-phosphorylating) activity | MF | 4 | 4 | 0.038528 | 1 | [3](file:///C:\Users\亚西\Desktop\BQ-PF20251106-原核转录2G-XJCD-6-潘飞老师-项目交付\Result\09_GO\Control-VS-Drug\Control-VS-Drug.GO_0043878_Ups.xlsx) | [1](file:///C:\Users\亚西\Desktop\BQ-PF20251106-原核转录2G-XJCD-6-潘飞老师-项目交付\Result\09_GO\Control-VS-Drug\Control-VS-Drug.GO_0043878_Down.xlsx) |
| GO:0034605 | cellular response to heat | BP | 4 | 4 | 0.039021 | 1 | [2](file:///C:\Users\亚西\Desktop\BQ-PF20251106-原核转录2G-XJCD-6-潘飞老师-项目交付\Result\09_GO\Control-VS-Drug\Control-VS-Drug.GO_0034605_Ups.xlsx) | [2](file:///C:\Users\亚西\Desktop\BQ-PF20251106-原核转录2G-XJCD-6-潘飞老师-项目交付\Result\09_GO\Control-VS-Drug\Control-VS-Drug.GO_0034605_Down.xlsx) |
| GO:0019333 | denitrification pathway | BP | 4 | 4 | 0.039088 | 1 | [2](file:///C:\Users\亚西\Desktop\BQ-PF20251106-原核转录2G-XJCD-6-潘飞老师-项目交付\Result\09_GO\Control-VS-Drug\Control-VS-Drug.GO_0019333_Ups.xlsx) | [2](file:///C:\Users\亚西\Desktop\BQ-PF20251106-原核转录2G-XJCD-6-潘飞老师-项目交付\Result\09_GO\Control-VS-Drug\Control-VS-Drug.GO_0019333_Down.xlsx) |
| GO:0006402 | mRNA catabolic process | BP | 4 | 4 | 0.039596 | 1 | [3](file:///C:\Users\亚西\Desktop\BQ-PF20251106-原核转录2G-XJCD-6-潘飞老师-项目交付\Result\09_GO\Control-VS-Drug\Control-VS-Drug.GO_0006402_Ups.xlsx) | [1](file:///C:\Users\亚西\Desktop\BQ-PF20251106-原核转录2G-XJCD-6-潘飞老师-项目交付\Result\09_GO\Control-VS-Drug\Control-VS-Drug.GO_0006402_Down.xlsx) |
| GO:0008940 | nitrate reductase activity | MF | 4 | 4 | 0.039747 | 1 | [3](file:///C:\Users\亚西\Desktop\BQ-PF20251106-原核转录2G-XJCD-6-潘飞老师-项目交付\Result\09_GO\Control-VS-Drug\Control-VS-Drug.GO_0008940_Ups.xlsx) | [1](file:///C:\Users\亚西\Desktop\BQ-PF20251106-原核转录2G-XJCD-6-潘飞老师-项目交付\Result\09_GO\Control-VS-Drug\Control-VS-Drug.GO_0008940_Down.xlsx) |
| GO:0000160 | phosphorelay signal transduction system | BP | 21 | 35 | 0.041763 | 1 | [10](file:///C:\Users\亚西\Desktop\BQ-PF20251106-原核转录2G-XJCD-6-潘飞老师-项目交付\Result\09_GO\Control-VS-Drug\Control-VS-Drug.GO_0000160_Ups.xlsx) | [11](file:///C:\Users\亚西\Desktop\BQ-PF20251106-原核转录2G-XJCD-6-潘飞老师-项目交付\Result\09_GO\Control-VS-Drug\Control-VS-Drug.GO_0000160_Down.xlsx) |


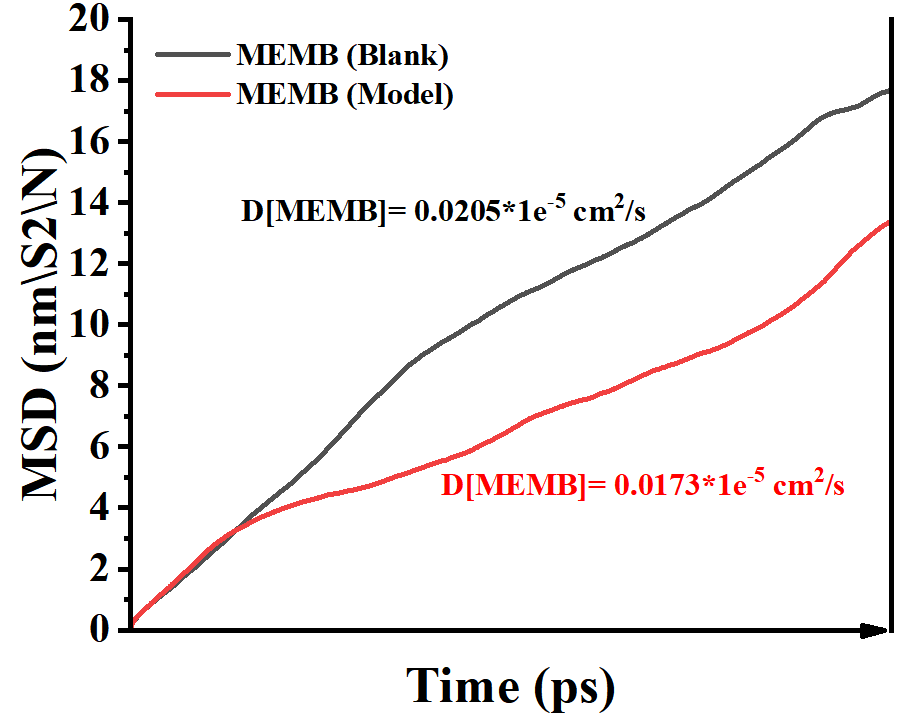


Figure S1. Comparison of mean square displacement (MSD) of MEMB (Blank) and MEMB (Model) systems.
